# Multilevel Interventions and Dental Attendance in Pediatric Primary Care: A Cluster Randomized Clinical Trial

**DOI:** 10.1001/jamanetworkopen.2024.18217

**Published:** 2024-07-09

**Authors:** Suchitra Nelson, Jeffrey M. Albert, David Selvaraj, Shelley Curtan, Hasina Momotaz, Gloria Bales, Sarah Ronis, Siran Koroukian, Johnie Rose

**Affiliations:** 1 Department of Community Dentistry, Case Western Reserve University School of Dental Medicine, Cleveland, Ohio; 2 Department of Population and Quantitative Health Sciences, Case Western Reserve University School of Medicine, Cleveland, Ohio; 3 University Hospitals Rainbow Center for Child Health & Policy, Cleveland, Ohio; 4 Department of Pediatrics, Case Western Reserve University School of Medicine, Cleveland, Ohio; 5 Center for Community Health Integration, Case Western Reserve University School of Medicine, Cleveland, Ohio

## Abstract

**Importance:**

Untreated tooth decay is disproportionately present among low-income young children. While American Academy of Pediatrics (AAP) guidelines require pediatric clinicians to implement oral health care, the effectiveness of these oral health interventions has been inconclusive.

**Objective:**

To test the effectiveness of multilevel interventions in increasing dental attendance and reducing untreated decay among young children attending well-child visits (WCVs).

**Design, Setting, and Participants:**

The Pediatric Providers Against Cavities in Children’s Teeth study is a cluster randomized clinical trial that was conducted at 18 pediatric primary care practices in northeast Ohio. The trial data were collected between November 2017 and July 2022, with data analyses conducted from August 2022 to March 2023. Eligible participants included Medicaid-enrolled preschoolers aged 3 to 6 years attending WCVs at participating practices who were enrolled at baseline (WCV 1) and followed-up for 2 consecutive examinations (WCV 2 and WCV 3).

**Interventions:**

Clinicians in the intervention group received both the practice-level (electronic medical record changes to document oral health) and clinician-level (common-sense model of self-regulation theory–based oral health education and skills training) interventions. Control group clinicians received AAP-based standard oral health education alone.

**Main Outcomes and Measures:**

Dental attendance was determined through clinical dental examinations conducted by hygienists utilizing International Caries Detection and Assessment System criteria and also from Medicaid claims data. Untreated decay was determined through clinical examinations. A generalized estimating equations (GEE) approach was used for both clinical examinations and Medicaid claims data.

**Results:**

Eighteen practices were randomized to either intervention or control. Participants included 63 clinicians (mean [SD] age, 47.0 [11.3] years; 48 female [76.2%] and 15 male [23.8%]; 28 in the intervention group [44.4%]; 35 in the control group [55.6%]) and 1023 parent-child dyads (mean [SD] child age, 56.1 [14.0] months; 555 male children [54.4%] and 466 female children [45.6%]; 517 in the intervention group [50.5%]; 506 in the control group [49.5%]). Dental attendance from clinical examinations was significantly higher in the intervention group (170 children [52.0%]) vs control group (150 children [43.1%]) with a difference of 8.9% (95% CI, 1.4% to 16.4%; *P* = .02). The GEE model using clinical examinations showed a significant increase in dental attendance in the intervention group vs control group (adjusted odds ratio, 1.34; 95% CI, 1.07 to 1.69). From Medicaid claims, the control group had significantly higher dental attendance than the intervention group at 2 years (332 children [79.6%] vs 330 children [73.7%]; *P* = .04) but not at 3 years. A clinically but not statistically significant reduction in mean number of untreated decay was found in the intervention group compared with controls (B = −0.27; 95% CI, −0.56 to 0.02).

**Conclusions and Relevance:**

In this cluster randomized clinical trial, children in the intervention group had better dental outcomes as was evidenced by increased dental attendance and lower untreated decay. These findings suggest that intervention group clinicians comprehensively integrated oral health services into WCVs.

**Trial Registration:**

ClinicalTrials.gov Identifier: NCT03385629

## Key Points

**Question:**

Is expanded theory-based oral health education and electronic medical record documentation vs standard education for clinicians effective in increasing dental attendance and decreasing untreated decay among young children?

**Findings:**

In this cluster randomized clinical trial of 18 pediatric primary care practices with 63 clinicians and 1023 parent-child dyads, clinical examinations demonstrated that children in the intervention group had 34% increased odds of dental attendance and clinically meaningful, lower untreated decay compared with controls. Medicaid claims data showed that children in the control group had increased odds of attendance but did not have lower untreated decay.

**Meaning:**

These findings suggest that clinicians receiving expanded oral health training can effectively counsel parents and increase timely dental care for children.

## Teaser

This cluster randomized clinical trial analyzed the effect of practice-level and clinicial-level interventions on dental attendance and untreated tooth decay among children with Medicaid insurance.

## Introduction

Nationally, 17.8% of 2- to 8-year old children in the US living below the federal poverty level have untreated dental caries (ie, tooth decay and cavities).^1^ Previous analyses of 3- to 6-year-old Medicaid-insured children from the cohort analyzed in this current study have shown an even higher prevalence of 29%.^2^ Receipt of preventive dental services among Medicaid-enrolled children nationally has risen from 42% to 48% between 2011 to 2018, but the national goal of 52% remains unmet.^3^

The disparate burden of dental caries among young children^4^ has prompted recommendations for primary care clinicians to provide preventive oral care for children 5 years and younger, including referral to a dentist.^5^ A 2023 systematic review^6^ by the US Preventive Services Task Force^7^ concluded that there is insufficient evidence for oral health (OH) screening, counseling, referrals, and preventive interventions performed by primary care clinicians in reducing dental caries. The few retrospective cohort studies that have examined the effectiveness of nondental clinicians delivering preventive OH services to young children enrolled in Medicaid have also been inconclusive.^8,9,10,11,12^

Common parent (or caregiver) misperceptions include a lack of awareness of the importance of baby teeth and a belief that cavities are always accompanied by pain or discomfort.^13^ Thus, an accurate understanding of the causes of caries and their prevention is required for parents to deploy self-management strategies and address their child’s OH needs. In one study,^14^ 2- to 5-year-old children were 3 times more likely to visit a dentist when advised by a primary health care clinician, but this study also reported that low-income families were less likely to be advised. Thus, proactive advice by primary care clinicians leverages evidence that parents and caregivers perceive health care clinicians to be a trusted source for health information.^15^

This cluster randomized clinical trial (cRCT) tested the hypothesis that a common-sense model (CSM) of self-regulation theory–based^16^ intervention at the practice and clinician levels would increase dental attendance among 3- to 6-year-old Medicaid-enrolled children compared with clinician only standard OH education based on American Academy of Pediatrics (AAP) curriculum. Secondarily, we investigated the effect of the intervention on reducing untreated decay in primary teeth.

## Methods

### Study Design

The Pediatric Providers Against Cavities in Children’s Teeth study is a cRCT with 2 parallel groups. The institutional review board of University Hospitals Cleveland Medical Center approved the study protocol (Supplement 1). Clinicians (pediatricians and nurse practitioners [NPs]) and parent- or caregiver-child dyads were study participants, and written informed consent was obtained from the participants in English. The trial adheres to the Consolidated Standards of Reporting Trials (CONSORT) reporting guideline.^17^

### Participants

The study included 18 primary care practices in northeast Ohio, their clinicians, and parent/caregiver-child dyads attending well-child visits (WCVs). Recruitment of clinicians and parent- or caregiver-child dyads occurred between November 2017 and August 2019 by the research staff; details have been published previously.^18^ Each dyad was followed for 3 consecutive WCVs (WCV 1 was baseline, with approximately annual follow-up at WCV 2 and WCV 3) between October 2018 and July 2022.

The cRCT inclusion criteria were (1) hospital-affiliated or independent practices with 20% or greater Medicaid-enrolled children that use electronic medical records (EMR); (2) pediatricians and NPs with a minimum of 2 patient-care days per week; and (3) children were 3 to 6 years old at the time of study enrollment, Medicaid insured, and without serious medical conditions preventing participation in dental screening. The parent or caregiver was required to be the child’s legal guardian, aged 18 years or older, comfortable speaking English, and have intent to reside in the study area throughout the study duration. All parent-child dyads were given a cash incentive of $40 for each completed visit as compensation for their time.

### Randomization and Blinding

Cluster randomization was performed by the biostatistician (J.M.A.) using restricted randomization to assign 9 practices to each group. The restricted randomization (based on a balance score) assured good balance in 3 practice-level variables: percentage of Medicaid-enrolled patients (20% to 40% vs >40%), ratio of patients to clinicians, and county (urban county vs other). Practices, clinicians, parents, and dental hygienists were blinded to the study group.

### Intervention

The practices in the intervention group received multilevel interventions. Clinicians received didactic education and skills training to learn about the chronicity of caries, communicate core OH facts to parents and caregivers at WCVs, and provide a prescription to visit the dentist together with a list of Medicaid-accepting dentists in the area. The curriculum based on the CSM framework was developed using existing training modules from the AAP as a starting point.^19^ A major difference between the intervention group’s expanded CSM theory–based education and the standard AAP-based curriculum was the emphasis of the former on the importance of baby teeth and their role in perpetuating and transferring cariogenic bacteria to permanent teeth. Practices incorporated 4 OH questions into the age-appropriate WCV note templates in each practice’s EMR. Clinicians received EMR training to document the OH encounter. Intervention group clinicians delivered core OH facts, gave a prescription to visit the dentist, and documented in the EMR regarding the OH encounter for all parents in this group. Details of the intervention are given in eTable 1 in Supplement 2.

The control group clinicians received non–theory-based AAP OH education and were asked to follow usual OH care recommended by AAP Bright Futures guidelines.^20^ No EMR documentation was required of control clinicians. Fidelity measures employed for the study have been previously reported.^13^

### Outcomes

For each child, 2 primary dental attendance outcome measures were created: clinical examination evidence of treatment (yes or no for any new restoration, extraction, or sealant), and administrative evidence of visits (yes or no) based on Medicaid claims data. Untreated decay was also assessed from clinical examinations. Reporting dental attendance separately was necessary due to primary (clinical examinations) and secondary (administrative data) data sources. The original plan was to follow children for 2 years, but WCVs were delayed due to the COVID-19 pandemic and, hence, clinical examinations occurred later, which necessitated looking at Medicaid claims data for up to 3 years. Therefore, for clinical examinations the primary outcome was cumulative dental attendance between baseline WCV 1 and the exit examination at WCV 3, and for Medicaid data it was cumulative dental attendance between baseline WCV 1 and at the end of 2 and 3 years.

Clinical visual examinations were conducted by trained dental hygienists at all 3 WCVs on an examination room table at each practice with disposable supplies. The dental hygienists were calibrated at baseline against a dentist expert in International Caries Detection and Assessment System (ICDAS) criteria, with a weighted κ of 0.67 to 0.83 for lesions and unweighted κ of 0.96 to 0.99 for fillings, sealants, and extractions indicating good to excellent reliability. A booster session followed a year later.

Dental attendance from clinical examinations was determined if 1 or more teeth had the any of the following at WCV 2 and/or WCV 3 that were previously not identified at baseline WCV 1: new restorations (ICDAS filling code ≥3, which includes tooth-colored restorations; amalgam; a stainless steel crown; or a gold, ceramic, or porcelain crown); extraction of 1 or more teeth; and new prevention (ICDAS filling code 1 and 2, which includes full or partial sealants) at WCV 2 and/or 3. For each child, a change in dental status between baseline and WCV 3 was calculated as the total number of teeth with restoration, extraction, or prevention not previously seen at baseline. From this, a binary variable (yes or no) was created for any dental visit (ie, preventive and restorative visit between WCV 1 and WCV 3). Untreated decay (ie, existing primary decayed teeth [dt]) was defined as ICDAS lesion code 3 or greater (localized to extensive decay) on 1 or more teeth. Both counts of teeth with these lesions and a binary variable (yes [dt ≥1] vs no [dt = 0]) were used for the analysis.

Medicaid claims data were obtained through a data use agreement between University Hospitals Cleveland Medical Center and the Ohio Department of Medicaid. From Medicaid claims files, *Current Dental Terminology 2020* codes used for billing were utilized to determine whether the child received preventive procedures (diagnosis, D0110-D0330; prevention, D1110-D1351; D4355) or restorative procedures (fillings or extractions, D2110-D2394; D1510-D1550; D2390-D4342; D7110-D7140; and D9420) (eTable 2 in Supplement 2). Dental attendance was collapsed into a binary variable (yes or no) for any dental visit, only preventive visits, or only restorative visits cumulatively between baseline to 3 years.

### Sociodemographic Variables

Baseline sociodemographic variables included child age, sex, race, ethnicity, parent age, and education collected through a self-reported parental questionnaire. The questions were adapted from the National Health and Nutrition Examination Survey^21^ and items were collapsed into categories for analysis as follows: child and parent sex (male or female), race (Black, White, and other [defined as American Indian or Alaska Native, Asian, Hawaiian or Pacific Islander, and multiracial]), ethnicity (Hispanic or non-Hispanic), age in months (children) or years (parents), and parent education (≤high school or >high school). Race and ethnicity were included as variables to account for disparities in outcomes.

### Statistical Analysis

The sample size, accounting for 0.04 within-practice correlation and 25% dropout rate, had 80% power to detect a difference in 2-year dental attendance rates of 18% (48% in the experimental group and 30% in the usual care group), based on a 2-sided .05 α-level Z test (with pooled variance). Missing data occurred due to missed examinations for clinical data and due to claims not received for some participants for Medicaid data. For the clinical data, we defined cumulative (or any) dental attendance up to WCV 3. Any attendance found at WCV 3 implied attendance through WCV 2. In addition, the number of untreated dt were determined at each WCV. The overall change in untreated decay was defined as the difference in dt counts from baseline WCV 1 to WCV 3. Cumulative dental attendance from the Medicaid data at 2 and 3 years was determined as follows: participants who had a dental claim within the 2- and 3-year interval were coded as yes and participants without a dental claim during the 2- and 3-year interval but had a baseline WCV claim were coded as no.

To assess the intervention effect on dental attendance, a generalized estimating equations (GEE) approach was used for both clinical examinations and Medicaid claims data. The intent-to-treat principle with respect to intervention assignment was followed. For the dental attendance using clinical examinations and the change in untreated dt outcomes, logit and identity links were used, respectively. An exchangeable working correlation structure for clustering of participants within practice was used for all these models. For the binary dental attendance outcomes using Medicaid claims, a logit link (ie, logistic regression) was used with an exchangeable working correlation structure. All models included an indicator for study group and covariates based on the scientific literature or child’s sex, race (collapsed as Black and all other race categories), age, parent education, and baseline untreated decay if found to be different between the study groups. Robust variance estimates (accounting for clustering within practice) were obtained. Estimated adjusted odds ratios (aORs) were obtained for binary outcomes, and an estimated mean difference was obtained for the change outcome. Tests for nonnull effects were performed using Wald χ^2^ tests, and 95% CIs were computed. Analyses were conducted using SAS Version 9.4 (SAS Institute).

Analyses assumed missing outcomes were missing at random.^22^ Randomness of missingness for the attendance outcome was examined using appropriate tests (χ^2^ or *t* tests) of the association of missingness with each covariate. The bivariate analysis found that missingness was only associated with child’s age (eTable 3 and eTable 4 in Supplement 2), but the mean child age was not different between the 2 study groups. As a sensitivity analysis, multiple imputation analysis was computed for clinically determined attendance because this was subject to substantially more missingness than attendance based on Medicaid claims. Imputations, providing 40 completed datasets, were obtained using the fully conditional specification^23^ including all the model variables, logistic regression for categorical variables, and linear regression with predictive mean matching for continuous variables. The GEE analysis was carried out for each dataset and results were combined using the Rubin method.^24^ Data analysis was conducted from August 2022 to March 2023.

## Results

Participants included 63 clinicians (mean [SD] age, 47.0 [11.3] years; 48 female [76.2%] and 15 male [23.8%]; 28 in the intervention group [44.4%]; 35 in the control group [55.6%]; 54 pediatricians [85.7%]; 9 NPs [14.3%]); and 1023 parent-child dyads (mean [SD] child age, 56.1 [14.0] months; 555 male children [54.4%] and 466 female children [45.6%]; 517 in the intervention group [50.5%]; 506 in the control group [49.5%]). In total, 1024 parent-child dyads from 9 intervention practices and 9 control practices were initially enrolled at baseline WCV 1, but 1 child in the control group had late exclusion for not fulfilling inclusion criteria (Figure). Retention rate was 79% (811 participants) and 74% (760 participants) at WCV 2 and 3, respectively. Medicaid claims were available for 872 enrolled children (85.2%). For longitudinal analysis of clinical data, 695 had both WCV 1 and 2 and 632 had WCV 1 and 3. The mean (SD) duration between WCV 1 to WCV 3 was 27.8 (5.3) months. Both study groups were balanced on all baseline sociodemographic characteristics except for Hispanic ethnicity. The mean number of primary and permanent teeth at baseline was similar between the intervention and control group (Table 1).

**Figure.**
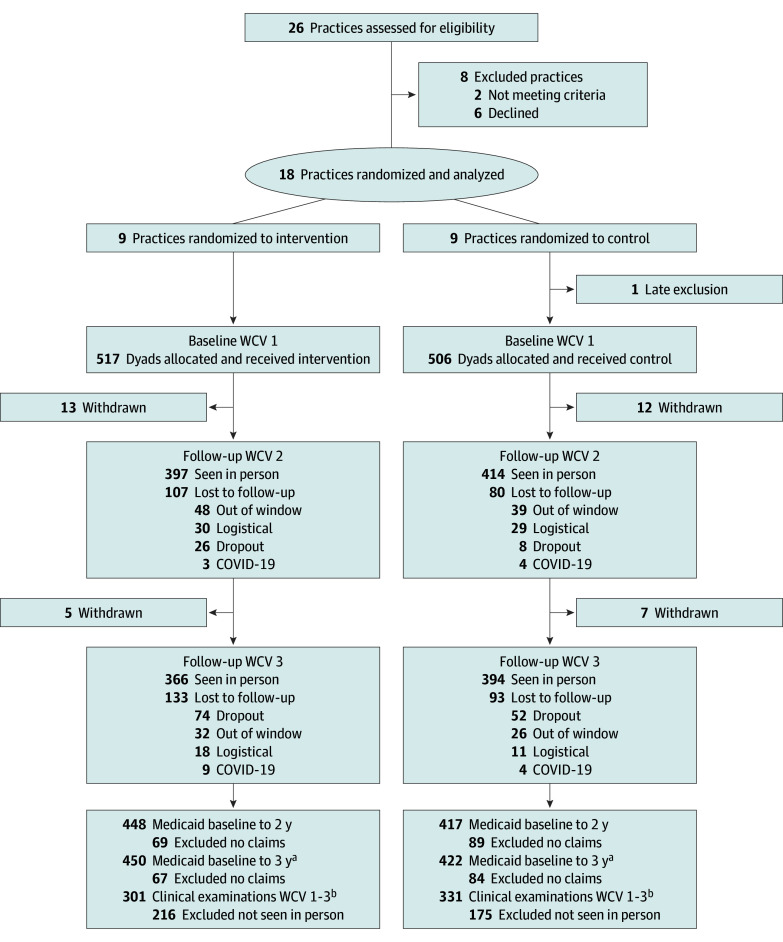
Study Flow Diagram ICDAS indicates International Caries Detection and Assessment System; WCV indictes well-child visit. ^a^Was in the Medicaid data 0 to 3 years after WCV 1. ^b^Had WCV 1 examination and WCV 2 or WCV 1 and WCV 3.

**Table 1.** Baseline Child and Parent or Caregiver Characteristics Between Study Groups

Variables^a^	Participants, No. (%)		
	Overall (N = 1023)	Intervention (n = 517)	Control (n = 506)
Child			
Race			
Black	451 (45.6)	226 (45.0)	225 (46.2)
White	441 (44.6)	232 (46.2)	209 (46.2)
Other^b^	97 (9.8)	44 (8.8)	53 (10.9)
Ethnicity			
Hispanic or Latino	76 (7.9)	28 (5.8)	48 (10.1)
Not Hispanic or Latino	882 (92.1)	457 (94.2)	425 (89.9)
Sex			
Female	466 (45.6)	228 (44.2)	238 (47.1)
Male	555 (54.4)	288 (55.8)	267 (52.9)
Age, mean (SD), mo	56.10 (14.0)	56.38 (14.34)	55.82 (13.72)
No. of primary teeth, mean (SD)	19.15 (1.84)	19.09 (1.89)	19.21 (1.79)
No. of permanent teeth, mean (SD)	1.22 (2.60)	1.29 (2.71)	1.15 (2.47)
Parent or caregiver			
Sex			
Female	920 (90.1)	463 (89.9)	457 (90.3)
Male	101 (9.9)	52 (10.1)	49 (9.7)
Race			
Black	438 (44.4)	223 (44.7)	215 (44.1)
White	497 (50.4)	256 (51.3)	241 (49.5)
Other^b^	51 (5.2)	20 (4.0)	31 (6.4)
Ethnicity			
Hispanic	51 (5.3)	20 (4.1)	31 (6.5)
Not Hispanic	913 (94.7)	466 (95.9)	447 (93.5)
Age, mean (SD), y	31.42 (7.48)	31.73 (7.89)	31.10 (7.02)
Education			
≤High school^c^	450 (44.7)	230 (45.5)	220 (43.8)
>High school^d^	557 (55.3)	275 (54.5)	282 (56.2)

^a^ Less than 6% of responses were missing for each of the variables.

^b^ Other consisted of American Indian or Alaska Native, Asian, Hawaiian or Pacific Islander, and multiracial.

^c^ Consisting of grades 7 to 11 and high school diploma or general education development.

^d^ Consisting of associate degree or some college, college or university degree, some graduate school, and graduate degree.

The intervention group had a significantly greater percentage of children with baseline untreated dt compared with the control group (165 children [32.0%] vs 133 children [26.3%]; *P* = .047) (Table 2). Children in the intervention group also had a significantly higher mean (SD) number of dt at baseline (0.97 [1.94] dt vs 0.72 [1.67] dt; *P* = .03). At the follow-up visits, the percentage of children with dt remained stable for the intervention group (133 children [38.8%] at WCV 2 and 117 children [38.7%] at WCV 3), but increased progressively for the control group from baseline (117 children [33.1%] at WCV 2 and 129 children [38.9%] at WCV 3) (Table 2). Between WCV 1 and 3, the mean (SD) untreated dt was 53% lower in the intervention group compared with the control group, but this finding was not significant (0.22 [2.10] dt vs 0.47 [2.12] dt; *P* = .13).

**Table 2.** Child Untreated Decay and Dental Attendance Between Study Groups–Bivariate Analysis

Variables	Overall (N = 1023)	Intervention (n = 517)	Control (n = 506)	*P* value^a^
Untreated decay (clinical examinations)				
Baseline WCV 1 participants, No.	1021	516	505	NA
Primary dt, mean (SD)	0.85 (1.82)	0.97 (1.94)	0.72 (1.67)	.03
Primary dt, No. (%)	298 (29.2)	165 (32.0)	133 (26.3)	.047
WCV 2 participants, No.	696	343	353	NA
Primary dt, mean (SD)	1.10 (2.13)	1.19 (2.28)	1.01 (1.96)	.27
Primary dt, No. (%)	250 (35.9)	133 (38.8)	117 (33.1)	.12
WCV 3 participants, No.	634	332	302	NA
Primary dt, mean (SD)	1.17 (2.09)	1.14 (2.12)	1.20 (2.07)	.75
Primary dt, No. (yes %)	246 (38.8)	117 (38.7)	129 (38.9)	.98
Between baseline and WCV 3 participants, No.	632	301	331	NA
Difference in primary dt, mean (SD)	0.35 (2.11)	0.22 (2.10)	0.47 (2.12)	.13
Difference in primary dt, mean (SD), %	0.10 (0.54)	0.08 (0.56)	0.11 (0.53)	.46
Dental attendance (clinical examinations): baseline to WCV 3 participants, No.	675	327	348	NA
Any dental visit, No. (yes %)	320 (47.4)	170 (52.0)	150 (43.1)	.02^b^
Preventive visit, No. (yes %)	117 (18.2)	69 (22.4)	48 (14.4)	.01^b^
Dental attendance (Medicaid data)				
Baseline to 2 y participants, No.	865	448	417	NA
Any dental visit, No. (%)	662 (76.5)	330 (73.7)	332 (79.6)	.04^b^
Preventive visit, No. (%)	635 (73.4)	312 (69.6)	323 (77.5)	.01^b^
Dental attendance: Medicaid data baseline to 3 y participants , No.	872	450	422	NA
Any dental visit, No. (%)	730 (83.7)	369 (82.0)	361 (85.5)	.16
Preventive visit, No. (%)	707 (81.1)	354 (78.7)	353 (83.6)	.06

Abbreviations: dt, untreated decayed teeth; NA, not applicable; WCV, well-child visit.

^a^ *P* values based on χ^2^ test, and *t* test.

^b^ Significance at α < .05.

The bivariate analysis from clinical data shows that dental attendance between baseline and WCV 3 was significantly higher in the intervention compared with control group in any dental visit (170 children [52.0%] vs 150 children [43.1%]; difference, 8.9%; 95% CI, 1.4%-16.4%; *P* = .02) and preventive visit (69 children [22.4%] vs 48 children [14.4%]; *P* = .01) (Table 2). There was a greater percentage of children that had administrative evidence for any dental attendance in the control compared with intervention group from baseline to 2 years (332 children [79.6%] vs 330 children [73.7%]; *P* = .04). By 3 years, most children had dental attendance (730 children [83.7%]), which was not significantly different between the groups.

The GEE model from clinical examinations shows that the intervention group had significantly higher estimated odds of dental attendance compared with controls between baseline to WCV 3 (aOR, 1.34; 95% CI, 1.07 to 1.69) (Table 3). However, the GEE Medicaid model showed there was significantly lower attendance among the intervention compared with control groups for baseline to 2 years (aOR, 0.71; 95% CI, 0.53 to 0.94), but this was not significant for the baseline to 3-year model (aOR, 0.77; 95% CI, 0.57 to 1.04) (Table 3). The intervention group had a clinically meaningful but not statistically significantly lower estimated change (ie, greater reduction) in mean number of dt compared with controls (β =  −0.27; 95% CI, −0.56 to 0.02) (Table 4). The bivariate analysis found that missingness was only associated with child’s age (eTable 3 and eTable 4 in Supplement 2), but the mean child age was not different between the 2 study groups. The multiple imputation (sensitivity) analysis for clinical dental attendance corroborated the results, showing a statistically significant increase in dental attendance at 3 years for the intervention vs control groups (aOR, 1.34; 95% CI, 1.01 to 1.77).

**Table 3.** Generalized Estimating Equation Model for Any Dental Attendance Using Clinical Examinations and Medicaid Claims

Variable	aOR (95% CI)^a^		
	Clinical baseline to well-child visit 3 (n = 651)	Medicaid baseline to 2 y (n = 826)	Medicaid baseline to 3 y (n = 832)
Intervention vs control	1.34 (1.07-1.69)	0.71 (0.53-0.94)	0.77 (0.57-1.04)
Age, mo	1.05 (1.03-1.06)	1.01 (1.00-1.02)	1.00 (1.0-1.01)
Black compared with all other races	1.07 (0.82-1.40)	1.14 (0.87-1.49)	1.02 (0.77-1.35)
Female vs male	0.95 (0.69-1.31)	0.97 (0.65-1.45)	0.97 (0.58-1.62)
≤High school vs >high school	1.10 (0.83-1.46)	0.91 (0.66-1.25)	0.98 (0.67-1.42)
Baseline primary dt (count)	1.32 (1.13-1.54)	1.00 (0.95-1.05)	0.97 (0.92-1.02)

Abbreviations: aOR, adjusted odds ratio; dt, untreated decayed teeth.

^a^ Binomial distribution.

**Table 4.** Generalized Estimating Equation Model for Untreated Decay Difference (Baseline to WCV 3) Using Clinical Data

Variable	Untreated decay count difference (baseline to WCV 3), β (95% CI)^a^
Participants, No.	608
Intervention vs control	−0.27 (−0.56 to 0.02)
Age, mo	−0.02 (−0.02 to −0.01)
Black compared with all other races	0.14 (−0.18 to 0.47)
Female vs male	−0.07 (−0.34 to 0.20)
≤High school vs high school	0.02 (−0.37 to 0.42)

Abbreviation: WCV 3, well-child visit 3.

^a^ Normal distribution.

## Discussion

To our knowledge, this is the first cRCT to test the effectiveness of multilevel OH interventions delivered by pediatricians and NPs in community pediatric practices. This trial shows the effectiveness of OH care provided by nondental clinicians, addressing the gaps in evidence detailed by the 2023 US Preventative Service Task Force review.^7^ The uniqueness of this trial is that we had availability of both clinical examination data and Medicaid claims to investigate dental attendance. Studies suggest that using claims data alone for follow-ups and event adjudication in clinical trials may not be as accurate as traditional approaches such as clinical data.^25,26^

The major findings from our trial are as follows. First*,* the annual dental attendance in both groups was 52% or greater, which is higher than the current estimates and meets the Centers for Medicare & Medicaid Services national goal for children,^3^ despite the disruptions caused by the COVID-19 pandemic. Second*,* the surprising conclusion from our study was that the clinical data highlights that children in the intervention group were receiving more comprehensive dental care (ie, they received significantly more sealants, fillings, and extractions documented by study hygienists, despite fewer visits based on administrative evidence for annual attendance). This timely dental care was concordant with our observation of a 0.27 lower mean number of untreated dt in the intervention group. Therefore, our findings of reduced decay in nearly one-third of the tooth can be clinically significant because existing early childhood caries is the single greatest predictor of future caries due to the cariogenic bacterial load^27,28^; studies also report that when more surfaces are affected (ie, increased severity), it increases caries in other primary and permanent teeth.^27,29,30^ We speculate that the counseling regarding baby teeth and resources to seek dental care delivered by clinicians in the intervention group resulted in qualitatively different care-seeking behaviors by parents (eg, actively pursuing dental care). The American Academy of Pediatric Dentistry specifies that early childhood caries remains a substantial problem; the potential for our intervention to lower untreated decay is timely,^31^ especially because the extent of untreated decay in our cohort was much higher than the national rate.^1^ Third*,* clinical examinations and Medicaid claims data gave conflicting results. However, our findings were similar to a systematic review of claims studies^32^ where the concordance between clinical judgement and claims data was low to average. In our study, children who had clinical examinations were seen at a WCV and therefore would have received the clinician intervention, but that was not the case for those with Medicaid claims data because not all of them had a claim for follow-up WCVs and therefore these parents would not have received the clinician intervention.

The findings from the clinical data are also concordant with the structured interviews conducted after trial completion^33^ where the intervention group clinicians reported that they gained knowledge about the importance of baby teeth and were confident in talking to parents about OH, whereas control clinicians more often described recommending oral hygiene practices. The CSM theory posits that understanding the chronicity of disease helps patients to self-manage their disease effectively.^34^ In this trial, clinician messaging in the intervention group was aimed to help parents understand that bacteria in cavitated baby teeth can easily attack the newly erupting permanent teeth and that timely dental attendance was important to prevent and/or arrest primary caries. Exit survey data was collected from parents regarding clinician delivery of the importance of baby teeth, symptoms of cavities, and importance of dental visits as was clinician documentation of OH questions in EMR. These data will be analyzed in the future to assess if they could have influenced dental care–seeking behaviors. Our finding of greater clinical evidence for restorations and sealants indicates that the clinicians in the intervention group most likely communicated this message effectively. A tool kit for dissemination has been developed to help clinicians with this new expanded CSM theory–based education that can be incorporated in professional websites.

The findings from Medicaid claims conflicted with those from the clinical data (ie, the controls had significantly more visits than the intervention group at 2 years but not at 3 years). However, the increased attendance among the control group was inconsistent with the clinical data because there was an increasing trend of untreated decay (ie, the control group started with less baseline untreated decay but increased progressively at each follow-up WCV). One possible reason for this finding could be that although children could have visited the dentist for screening, they may not have followed through with the necessary dental treatments. Part of this discrepancy also may be related to the purpose of claims data because they are made for administrative and reimbursement purposes, and may not accurately reflect, in this case, the content of those encounters. Similarly, they are not generated for clinical or research goals.^35^ Clinical trials that solely use Medicaid claims should consider validation with clinical data to support their outcomes.

### Limitations

There are limitations to this trial. First, follow-up losses at WCVs lowered the analysis sample size. In both clinical and Medicaid data, the only difference between participants and nonparticipants was child’s age (eTable 3 and eTable 4 in Supplement 2), but with adjustment for age in the GEE models, it is unlikely to alter the results. Second, Medicaid claims can overestimate dental attendance (eg, a child could have had a diagnostic radiograph without receiving any further dental care and this would still count as attendance). Dental caries diagnostic codes are not routinely used by dentists and reported in claims data except for procedure codes, and, therefore, if solely using claims data, it would not be possible to determine if a child with baseline caries actually received the needed dental care. Third, clinical data, on the other hand, will underestimate dental attendance (ie, it would not be possible for hygienists to detect cleanings, radiographs, and fluoride varnish applications through visual examinations).

## Conclusions

In this cRCT, young patients of pediatric clinicians with expanded theory-based education who delivered key OH facts about chronicity of caries at WCVs had increased restorative and preventive dental care and lower untreated decay. These findings suggest that clinicians receiving expanded oral health training can effectively counsel parents and increase timely dental care for children.

## Notes

Supplement 1.
Trial Protocol

Supplement 2. **eTable 1.** Details of Multi-Level Intervention **eTable 2.** Assumptions for Coding the Medicaid Claims Data **eTable 3.** Differences Between Participants and Non-Participants From Clinical Data (Baseline to WCV3) **eTable 4.** Differences Between Participants and Non-Participants From Medicaid Data (Baseline to 3 Years)

Supplement 3.
Data Sharing Statement
