# Supplementary material for: Multilevel Interventions and Dental Attendance in Pediatric Primary Care: A Cluster Randomized Clinical Trial
Source: JAMA Netw Open. 2024 Jul 9;7(7):e2418217. doi: 10.1001/jamanetworkopen.2024.18217 (PMC11234234; doi:10.1001/jamanetworkopen.2024.18217)
Supplement: Supplement 1. — Trial Protocol [file jamanetwopen-e2418217-s001.pdf]

Multi-Level Interventions to Reduce Caries Disparities in Primary Care Settings (Project PACT)

NIDCR Protocol Number: 17-082-E

NIDCR Grant Number: UH3DE025487

Principal Investigator: Dr. Suchitra Nelson

Trial Registration: clinicaltrials.gov (NCT03385629)

Draft or Version Number: 9.0

4 April 2022

CONFIDENTIAL No part of it may be transmitted, reproduced, published, or used without prior written authorization from the Principal Investigator.

TABLE OF CONTENTS

	PAGE
**TABLE OF CONTENTS**	2
**2 INTRODUCTION: BACKGROUND INFORMATION AND SCIENTIFIC RATIONALE**	10
2.1 Background Information	10
2.2 Rationale	12
2.3 Potential Risks and Benefits	13
2.3.1 Potential Risks	13
2.3.2 Potential Benefits	13
**3 OBJECTIVES**	14
3.1 Study Objectives	14
3.2 Study Outcome Measures	14
3.2.1 Primary Outcome Measures	14
3.2.2 Secondary Outcome Measures	14
3.2.3 Mediators, Moderators, Confounders, & Implementation Measures	15
**4 STUDY DESIGN**	16
4.1 Study participants	16
4.2 Model and Design	17
**5 STUDY ENROLLMENT AND WITHDRAWAL**	20
5.1 Subject Inclusion Criteria	20
5.2 Subject Exclusion Criteria	20
5.3 Strategies for Recruitment and Retention	20
5.4 Treatment Assignment Procedures	22
5.4.1 Randomization Procedures	22
5.4.2 Masking Procedures	23
5.5 Subject Withdrawal	23
5.5.1 Reasons for Withdrawal	23
5.5.2 Handling of Subject Withdrawals or Subject Discontinuation of Study Intervention34	23
5.6 Premature Termination or Suspension of Study	24
**6 STUDY INTERVENTION**	25

6.1 Study Behavioral or Social Intervention(s) Description 25
6.2 Administration of Intervention 26
6.3 Procedures for Training Interventionists and Monitoring Intervention Fidelity 27
6.3.1 Procedures for Training Interventionists 27
6.3.2 Procedures for Monitoring Intervention Fidelity 27
6.4 Assessment of Provider Compliance with Study Intervention 28
7 STUDY SCHEDULE 29
7. 1 Provider 29
7.1.1 Baseline Visit: WCV #1 29
Before WCV #1 (T_{-1}: Day -30 to -1) 29
At WCV #1 (T_0: Day 0) 29
7.1.2 Follow-up Visit: WCV #2 30
Before WCV #2 (T_2: Day 364 ± 90) 30
At WCV #2 (T_3: Day 365 ± 90) 30
7.1.3 Final Visit: WCV #3 30
At WCV #3 (T_6: Day 730 ± 90) 30
7.1.4 Provider Withdrawal Visit 30
7.2 Parent/Caregiver and Child 31
7.2.1 Baseline Visit: WCV #1 31
At WCV #1 (T_0: Day 0) 31
Before Provider Encounter 31
During Provider Encounter in the Exam Room 31
After Provider Encounter in the Exam Room/Waiting Area 32
After WCV #1 (T_1) 32
7.2.2 Follow-up Visit: WCV #2 32
At WCV #2 (T_3: Day 365 ± 90) 32
Before Provider Encounter in the Exam Room 32
During Provider Encounter in the Exam Room 32
After Provider Encounter in the Exam Room/Waiting Area 32
After WCV #2 (T_4): 33

7.2.3 Final Visit: WCV #3 33
At WCV #3 (T_6: Day 730 ± 90) 33
Before Provider Encounter in the Exam Room 33
During Provider Encounter in the Exam Room 33
After Provider Encounter in the Exam Room/Waiting Area 33
7.2.4 Parent/Caregiver and Child Withdrawal Visit 34
7.2.5 Parent/Caregiver and Child Unscheduled Visit 34
8 STUDY PROCEDURES / EVALUATIONS 35
8.1 Study Procedures/Evaluations 35
8.1.1 Child-level 36
8.1.2 Parent/caregiver 38
8.1.3 Provider 38
8.1.4 Practice 39
9 ASSESSMENT OF SAFETY 39
9.1 Specification of Safety Parameters 39
9.1.1 Unanticipated Problems 40
9.1.2 Adverse Events 40
9.1.3 Serious Adverse Events 40
9.2 Time Period and Frequency for Event Assessment and Follow-Up 40
9.3 Characteristics of an Adverse Event 41
9.3.1 Relationship to Study Intervention 41
9.3.2 Expectedness of SAEs 41
9.3.3 Severity of Event 41
9.4 Reporting Procedures 41
9.4.1 Unanticipated Problem Reporting to IRB and NIDCR 41
9.4.2 Serious Adverse Event Reporting to NIDCR 43
10 STUDY OVERSIGHT 45
11 CLINICAL SITE MONITORING 46
12 STATISTICAL CONSIDERATIONS 47
12.1 Study Hypotheses 47

12.2 Sample Size Considerations 47
12.4 Final Analysis Plan 48
13 SOURCE DOCUMENTS AND ACCESS TO SOURCE DATA/DOCUMENTS 51
14 QUALITY CONTROL AND QUALITY ASSURANCE 53
14.1 Definitions 53
14.2 Study Intervention and Study Questionnaires 53
14.2.1 Quality Assurance Procedures 53
14.2.2 Quality Control Procedures 54
15 ETHICS/PROTECTION OF HUMAN SUBJECTS 56
15.1 Ethical Standard 56
15.2 Institutional Review Board 56
15.3 Informed Consent Process 56
15.4 Subject Confidentiality 56
16 DATA HANDLING AND RECORD KEEPING 58
16.1 Data Management Responsibilities 58
16.2 Data Capture Methods 58
16.3 Types of Data 58
16.4 Schedule and Content of Reports 71 59
16.5 Study Records Retention 60
16.6 Protocol Deviations 72
17 PUBLICATION/DATA SHARING POLICY 75
18 LITERATURE REFERENCES 76

LIST OF ABBREVIATIONS

AAP	American Academy of Pediatrics
AAPD	The American Academy of Pediatric Dentistry
CC	Coordinating Center
CME	Continuing Medical Education
CFR	Code of Federal Regulations
CRF	Case Report Form
CSM	The Common-Sense Model of Self-Regulation
CTSC	Clinical and Translational Science Collaborative
DMFT	Decayed, Missing, Filled Teeth
EDC	Electronic Data Capture
EMR	Electronic Medical Record
FWA	Federal wide Assurance
GAO	U.S. Government Accountability Office
GCP	Good Clinical Practice
HHS	The U.S. Department of Health and Human Services
ICH	International Conference on Harmonisation
IPQ-RD	The Illness Perception Questionnaire – Revised for Dental
IRB	Institutional Review Board
MOC	Maintenance of Certification
MOP	Manual of Procedures
N	Number (typically refers to participants)
NIDCR	National Institute of Dental and Craniofacial Research
NIH	National Institutes of Health
OH	Oral Health
OHRP	Office for Human Research Protections
PACT	Protecting All Children's Teeth
PI	Principal Investigator
POHS	Preventive Oral Health Services
PTF	Practice-Tailored Facilitation
QA	Quality Assurance
QC	Quality Control
QI	Quality Improvement
RCC	Rainbow Care Connection
RCT	Randomized Clinical Trial
SAE	Serious Adverse Event/Serious Adverse Experience
SCT	Social Cognitive Theory
UHCMC	University Hospitals Cleveland Medical Center
UP	Unanticipated Problem
USPSTF	US Preventive Services Task Force
WCV	Well-Child Visit

PROTOCOL SUMMARY

Title: Multi-Level Interventions to Reduce Caries Disparities in Primary Care Settings

Précis:

The study is a multi-site, multi-level, and multi-component cluster randomized clinical trial (RCT) to address poor dental utilization (attendance) and untreated caries among 3-6 year old Medicaid enrolled children attending well-child visits (WCV) in primary care settings. The focus is on addressing factors (determinants) at the socio-ecological levels of the child's environment: provider (pediatrician and nurse practitioner), practice/organization level, and parent/caregiver level. Eighteen practices will be randomized to 2 arms: A) bundled multi-level intervention consisting of: 1. training medical providers in the Common-Sense Model of Self-Regulation theory-based education so that the provider delivers to the parent/caregiver the following: i) Core oral health facts about dental caries, and ii) prescription to visit the dentist and a list of dentists accepting Medicaid; 2. Integration of oral health assessments into EMR for the provider to document in the child's medical record; versus B) Control arm of medical providers receiving the American Academy of Pediatrics (AAP) based oral health education and providing usual AAP-based care for oral health. Each arm will consist of 9 practices. Children will be followed for 24 months or the completion of 3 consecutive WCV's to determine dental utilization and changes in oral health status.

Objectives:

Primary:

To examine the effectiveness of theory-based behavioral (provider-level) and implementation (practice-level) bundled interventions versus enhanced usual care (AAP based oral health education) delivered by providers at WCVs in increasing dental attendance among 3-6 year old Medicaid-enrolled children.

Secondary:

1) Assess the effectiveness of interventions on secondary outcomes (e.g. development of new caries, changes in oral hygiene, oral health quality of life, frequency of sweet snacks and beverages, cost).
2) Assess potential mediators and moderators to investigate the pathways through which the multi-level interventions affect child primary and secondary outcomes.
3) Assess the adoption, reach, fidelity, and maintenance of providers and practices that affect child primary and secondary outcomes.

**Population:**	Study participants: Pediatricians/Nurse Practitioners (n = 67) and child and parent/caregiver dyads (n = 1024) will be recruited from 18 primary care practices with ≥ 20% Medicaid eligible children. Practices are selected from Rainbow Care Connection (RCC), a pediatric Medicaid accountable care organization that is part of the Rainbow Research Network (Clinical and Translational Science Collaborative [CTSC] supported practice-based pediatric research network), University Hospitals Cleveland Medical Center. The recruited practices have 60 pediatricians and 7 nurse practitioners who are 73% female and 27% male. We expect child and parent/caregiver demographics to be similar to Census and Medicaid data from northeast Ohio where Medicaid-enrolled children are 61% African American, 32% Caucasian, 5% Hispanic, and 2% other.
**Phase:**	Phase III
**Number of Sites:**	18 primary care practice sites
**Description of Intervention:**	The bundled intervention will impact providers, the practice and the parent/caregiver. The Common-Sense Model of Self-Regulation (CSM)-based education and skills training for the pediatrician or nurse practitioner are designed to educate them regarding chronicity of caries and teach them how to communicate core oral health facts to the parent/caregiver, provide a prescription to take the child to the dentist together with a list of area dentists who accept Medicaid, and document oral health in the EMR. The parent/caregiver will receive the CSM-based facts from the providers and should take their child to the dentist and improve oral health behaviors in the home. The practice level intervention will be enhancements to the EMR system to include oral health status for quality improvement of records and to use for follow-up with the parent/caregiver at subsequent well child visits.
**Study Duration:**	Approximately 36 months
**Subject Participation Duration:**	Parent/Caregiver = 24 months *or completion of 3 WCV's*<br>Providers = 24 months or completion of 3 consecutive WCV's of enrolled patients. Additionally, all providers will complete a follow-up structured interview and debriefing session.
**Estimated Time to Complete Enrollment:**	9 months

Schematic of Study Design:

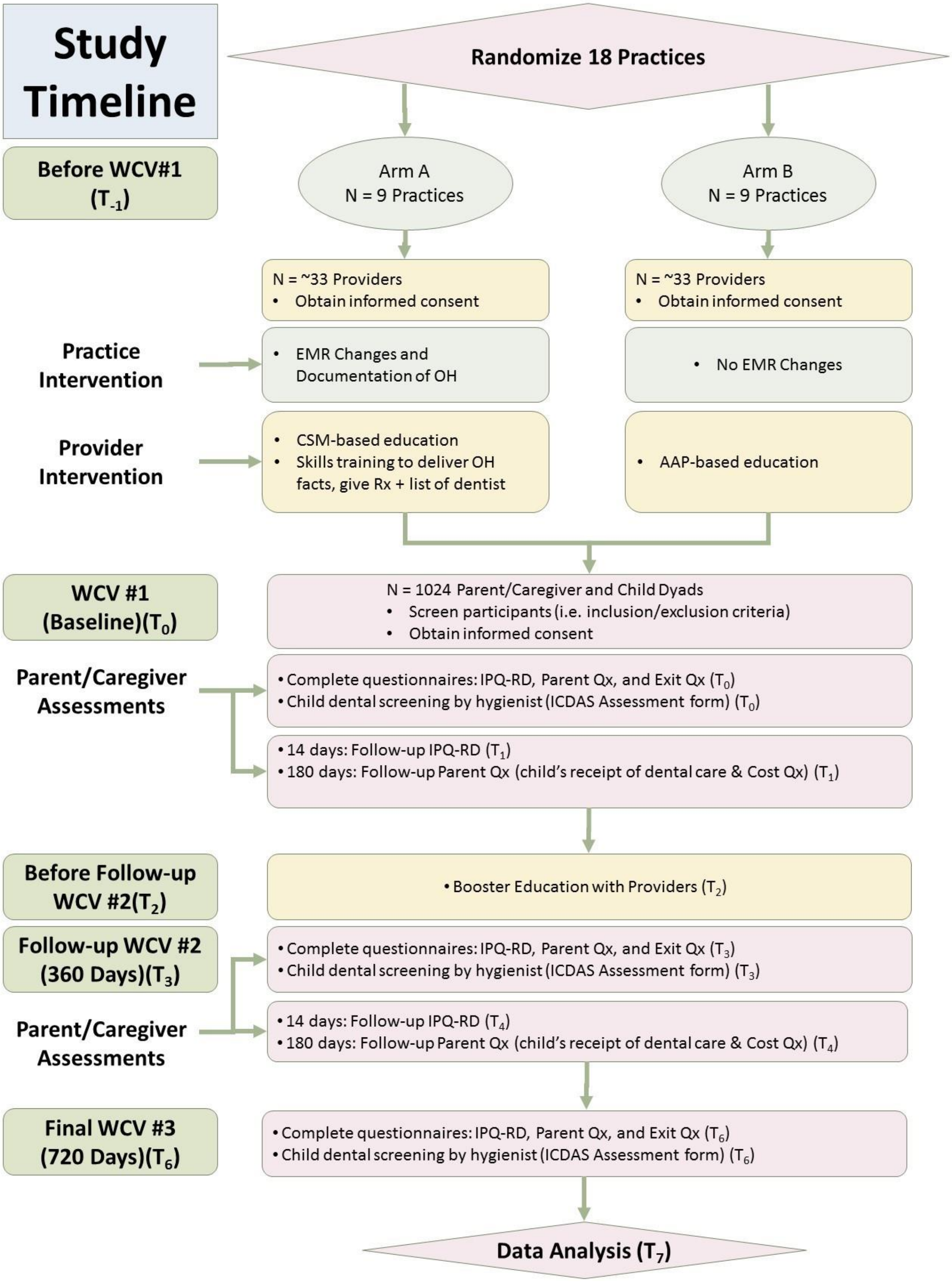

2 INTRODUCTION: BACKGROUND INFORMATION AND SCIENTIFIC RATIONALE

2.1 Background Information

Dental caries (tooth decay) is the leading chronic childhood disease.[1] Its prevalence in the primary dentition among 2-8 year old children increased from 35% (1988-1994) to 37% (2011- 2012) and failed to meet Healthy People objectives.[2] Although caries experience and untreated caries among 2-8 year old children have decreased nationally, poor and minority children still have a higher burden of disease compared to their affluent counterparts.[3] In Cleveland, our community-wide study indicated an untreated primary caries rate of 42%,[4] much higher than the national average of 14%.[5] Medicaid claims data from 2007 and 2008 indicates that median dental utilization among Medicaid-enrolled children was only 33-37%.[6,7] Recent self-reported surveys indicate that 50.6% of Medicaid-enrolled children compared to 69.0% of non-Medicaid enrolled children visited the dentist in the last six months.[8] For 13% of Medicaid-enrolled young children, their first dental visit was for emergency care.[9] There is clearly a dental care gap among 3-5 year-old Medicaid-enrolled children with 39% having had a dental visit,[6] despite anticipatory guidance for preventive dental visits starting from age 1,[10] while 67% (3-6 year olds) received a well-child visit.[11]

Due to the disparate burden of caries among young children,[2] primary care pediatric settings have been recommended to provide preventive oral care including referral to a dentist. But, a recent systematic review by the U.S. Preventive Services Task Force (USPSTF) to update its recommendation for medical primary care clinicians concluded that there is lack of evidence on effectiveness of parent/caregiver educational interventions and primary care referral to dentists.[12]

The few retrospective cohort studies that have examined the effectiveness of non-dental primary care providers delivering preventive oral health services (POHS: such as screening exams, dentist referral, fluoride varnish) to young Medicaid-enrolled children have resulted in lower decayed missing filled teeth (dmft) in kindergarteners who received POHS compared to those who did not.[13] The number of dmft at age 5 was similar regardless of the type of provider (primary care vs. dentist) delivering POHS.[14] Distance from physician-based POHS did not impact the likelihood of the child having a medical visit, while distance from a dental practice lowered dental utilization.[15]

One recent study found that caries-related expenditures (using Medicaid claims data from Alabama) among children <6 years were significantly higher for those receiving dentist delivered early preventive care versus those not receiving early care, while there was no difference between receiving early preventive care from a primary care provider versus not.[16] But, Milgrom and Cunha-Cruz in a letter to the editor regarding Blackburn et al.'s study,[17,16] point out that there are several methodological limitations and that the efficacy of fluoride varnish is dependent on being delivered according to a periodic schedule that can be affected by missed well-child visits which may have impacted their study conclusions. Thus, our proposed randomized clinical trial has the potential to address the limitations of prior retrospective studies in terms of testing behavioral interventions in pragmatic settings with careful control for confounders and longitudinal follow-up of children with clinical and claims data. The interventions being tested also offer the opportunity to lower untreated decay and increase dental utilization in 3-6 year-old Medicaid-enrolled children, two outcomes which were not able to be achieved in prior retrospective studies.[13-15] Additionally, in dental settings the effectiveness of interventions with

oral health (OH) education or motivational interviewing have been inconclusive and these interventions have had limited success in eliminating or reducing caries disparities in young children.[18-24]

The literature is clear that most low-income parent/caregivers see dental diseases as acute to be responded to only when there is pain or visible decay.[25] One approach shown to be useful in changing this reasoning process and for self-management of chronic medical conditions is the Common-Sense Model of Self-Regulation (CSM).[26,27] The CSM is a psychological approach where individuals create a mental representation (or perception) of their illness based on the abstract and concrete sources of information available to them. Six key domains guide the cognitive illness representation: identity, cause, consequence, timeline, cure or controllability, and illness coherence. The CSM being a "parallel-processing" model requires an emotional domain since individuals use past illness experience, cognitive, and emotional representation to self-manage their illness.[28] In this manner the CSM is unique from other social cognitive and health behavior theories.[29] A negative or positive illness perception is formed from three sources of information:[30] cultural knowledge of illness; information from external environment such as doctor/dentist or parent; and illness threat from past and current experiences with the disease. Based on the CSM, the revised illness perception questionnaire (IPQ-R) was developed[28] and adapted for various chronic illnesses with good reliability and validity.[31-36] Illness perception has been demonstrated empirically in other medical and psychological illnesses to predict health outcomes,[37-40] and in development of behavioral interventions to change perception and improve health outcomes.[41-43] Recently, our research team has shown that caregivers who believed baby teeth do not matter had significantly less accurate illness perception of dental caries compared to caregivers who believed baby teeth do matter utilizing the illness perception questionnaire revised for dental (IPQ-RD) that the team developed and validated.[44] So, CSM-based behavioral interventions have the potential to modify caregiver caries perception, and improve dental utilization for young children.

This multi-site, multi-level, and multi-component cluster randomized clinical trial (RCT) will assess dental utilization among children following oral health interventions in the primary care setting. The intervention mapping framework was used to develop the theory-based multi-level interventions at the provider and practice level.[45] Drawing on emerging concepts from the medical literature, the common-sense model of self-regulation (CSM) was used as the underlying theory for the provider-level interventions. Traditionally, oral health education has provided disjointed factual knowledge at the parent/caregiver level. In this study, providers are first trained in the organized CSM-based education to understand the chronicity of caries, and then taught to use the CSM framework to deliver key oral health facts to parent/caregivers with the goal of improving their cognitive and emotional representation with a chain of reasoning of the caries process. Other supporting theories such as the Social Cognitive Theory (SCT) are also used to improve the skills and self-efficacy at the provider level to communicate the oral health facts.[46] Providers will develop skills in 5 areas: 1) introduce the topic of dental care in routine clinical encounters, 2) strategies to effectively provide brief advice, 3) strategies to elicit and address parent/caregiver concerns in a nonconfrontational manner, 4) provide a prescription for the child to go to the dentist and a list of area Medicaid-accepting dentists, and 5) document oral health encounter in EMR. At a practice level, integration of OH documentation in EMR may be influenced by practice attributes and champion/s in accordance with Roger's diffusion of innovation theory.[47,48]

While the focus is on addressing factors at three levels of the child's environment (practice, provider, parent/caregiver, the multilevel experimental study interventions are at the provider (behavioral) and practice (implementation) levels. The parent/caregiver will be the recipients of the provider- and practice- level study interventions.

Provider (Pediatrician or Nurse Practitioner): (1) Providers will receive theory-based training to improve knowledge and skills using CSM and SCT frameworks; (2) Providers will communicate to parent/caregiver the CSM-based oral health facts, and provide prescription to go to the dentist + a list of Medicaid accepting dentists.

Parent/caregiver: Parents/caregivers will receive from the provider, CSM-based oral health facts and a prescription + list of Medicaid- accepting dentists to increase the child's dental attendance and improve oral health of the child, including oral hygiene behaviors in the home.

Practice (Pediatric): Practices will integrate documentation of oral health status in the EMR for quality improvement and to inform the providers of the child's oral health status and previous CSM-based oral health facts (maintenance of the intervention).

2.2 Rationale

Ten million U.S. preschool and school-age children have untreated caries and profound disparities exist by race, socioeconomic status, and geographic region. Our studies in Cleveland have found that school dental screenings have not been effective in stimulating dental attendance despite eligibility under the Affordable Care Act and coverage through Medicaid.[4] Our pilot study results among 86 three to six year old Medicaid-enrolled children indicate that 36% (31 out of 86) had untreated dental caries (cavitation), much higher than the national average of 18%.[3]

There are three core parent (or caregiver) issues: (1) awareness of importance of caries-free primary teeth; (2) understanding dental caries as a chronic disease that can be present even without symptomatic tooth pain; (3) navigating resources for dental care access. Thus, an accurate perception of dental caries is required for parental self-management strategies to take care of their child's oral health needs. Also important is that parent/caregivers receive consistent OH facts from primary care settings, where young children are most often seen. In one study, two to five year old children were 3 times more likely to visit a dentist when advised by a primary health care provider, but this study also reported that low-income families were less likely to be advised.[49]

Interventions that improve dental attendance among disparate populations are likely to reduce caries disparities. In this study, parents/caregivers are recipients of the intervention through a dissemination channel from providers to parents/caregivers. Parents/caregivers are not the direct target of the intervention, but indirectly benefit through the provider. This active dissemination strategy builds on evidence that parents/caregivers perceive healthcare providers to be trusted resources for health information. By enhancing dental knowledge and skills of the pediatric providers through the CSM-based education, interactive practice sessions with standardized patients, and training in EMR documentation, we hypothesize parents/caregivers will receive this new information along with coping and action supports (e.g., prescription to the dentist, list of Medicaid accepting dental providers) from the child's

provider. As a result, we hypothesize that providers with improved knowledge regarding the chronicity of caries and appropriate skills training will deliver consistent reinforcing oral health facts to parents (at annual WCVs) that will result in increased dental utilization for their child compared to those providers delivering usual care based on AAP education.

There are three pathways to dissemination of evidence-based interventions: The first pathway which is the most common is the "direct to practice" through the development of an exportable package or toolkit distributed through professional or public-health organizations; The second pathway involves "policy to practice" through establishment of policies to be followed by organizations or communities; The third pathway is systematic "diffusion research" to assess the effectiveness of different dissemination methods, and one that is least frequently used. A qualitative study among primary care providers indicated that health professionals require a tool kit that is demonstrated to be effective, easy to tailor and apply, has a table of contents to navigate to the relevant content, infrastructure/organizational support, and availability of support from practice facilitators or other experts. Further, tool kits can be an effective knowledge transfer strategy. Few theory-based tool kits exist for oral health interventions in primary care, therefore to address this gap we will develop a tool kit according to the first pathway utilizing the evidence from our cluster randomized clinical trial and the qualitative findings.

2.3 Potential Risks and Benefits

2.3.1 Potential Risks

Children: Children may experience some discomfort during the screening exam from keeping their mouths open for up to two minutes. There is also a chance that confidential information can be lost but we will use every precaution to protect the information we collect.

Parents/Caregivers: Some people are uncomfortable answering questions about themselves for research. We will try to make participants feel as comfortable as possible. There is also a chance that confidential information can be lost but we will use every precaution to protect the information. The oral health portion will add increased time to the well child visit that may impact parent/caregiver schedule and parking fees (if required to pay for parking).

Providers: Some people may feel uncomfortable being audiotaped or observed by study staff. We will try to make providers feel as comfortable as possible. There is also a chance that confidential information can be lost but we will use every precaution to protect the information we collect. As a result of participating in this study, there would be additional time added to the well-child visit that can potentially lead to some loss of productivity for providers.

2.3.2 Potential Benefits

Child participants may benefit by learning how to take care of his/her teeth and/or from their parent/caregiver learning about oral health. Parents/caregivers will receive information about taking the child to the dentist for preventive visits.

Providers may benefit from participating in the study by learning about oral health facts, integrating an oral health protocol into practice routines, and best practices for communicating oral health facts to parents/caregivers. EMR Documentation of oral health will help the providers to follow-up with the parent at the next well-child visit and be useful for practice quality improvement.

3 OBJECTIVES

3.1 Study Objectives

Primary Objectives:

1) To examine the effectiveness of theory-based behavioral (provider-level) and implementation (practice-level) bundled interventions versus enhanced usual care (based on AAP oral health education) delivered by providers at WCVs in increasing dental attendance among 3-6 year old Medicaid-enrolled children.

Secondary Objectives:

1) Assess the effectiveness of interventions on secondary outcomes (e.g. development of new caries, changes in oral hygiene, oral health quality of life, frequency of sweet snacks and beverages, cost).
2) Assess potential mediators and moderators to investigate the pathways through which the multi-level interventions affect child primary and secondary outcomes.
3) Assess the adoption, reach, fidelity, and maintenance of providers and practices that affect child primary and secondary outcomes.

3.2 Study Outcome Measures

Primary and secondary effectiveness outcomes will be assessed at the child level.

3.2.1 Primary Outcome Measures

The primary outcome will be dental attendance (receipt of dental care as yes/no, and number of visits). The primary outcome will be determined through a hierarchical process where Medicaid dental claims data (Current Dental Terminology (CDT) codes for preventive or restorative procedures in the past 12 months) would be the primary measure, followed by assessments through clinical dental screenings (restoration or extraction between WCVs #1 and #2, and between WCVs #2 and #3), conducted by calibrated examiners (See Section 14.2).

3.2.2 Secondary Outcome Measures

Secondary outcomes will be assessed through clinical dental screenings for new primary and permanent decayed and filled teeth (dft/DFT: number of new cavitated lesions or restorations on teeth that were previously sound and number of new restorations on teeth that were previously decayed at the baseline WCV), questionnaire responses for oral hygiene (frequency of tooth brushing), diet (frequency of sweet food and drinks), and oral health-related quality of life (ECOHIS, numeric score).[50] The direct and indirect dental costs will be assessed through various sources (See Table 8.1.2 for more details on cost elements and sources).

3.2.3 Mediators, Moderators, Confounders, & Implementation Measures

Mediators, moderators, confounders, and implementation measures will be assessed at the parent, provider and practice levels. The interventions are likely to influence mediators at the practice level (EMR documentation and practice readiness, climate, attitude) and at the provider level (oral health knowledge and self-efficacy). Subsequently, providers improved oral health knowledge and communication of oral health facts to parent is likely to influence parent-level mediators (illness perception and parent self-efficacy to take the child to the dentist). The moderators for the relationship between the interventions and the primary/secondary outcomes are parental socio-demographics, health literacy and social support and the confounders are child medical illness. Provider socio-demographics can also be a moderator to this relationship. See Sections 4 (Figure 4.2.1) for more details on mediators, moderators and confounders and Table 8.1.1 for implementation measures and for more details regarding how these measures will influence the primary and secondary outcomes.

3.3 Additional Objective with Supplement

To evaluate the process and outcomes data from the implementation of the cluster randomized clinical trial, together with additional provider qualitative data on barriers/enablers (using Roger's Diffusion of Innovation framework) collected during this revision for the development of a toolkit for dissemination to stakeholders.

As part of the supplement, providers will complete a follow-up structured interview and debriefing session.

4 STUDY DESIGN

This study will utilize a multi-site, multi-level, and multi-component cluster randomized clinical trial design (Phase III) to assess interventions intended to address poor dental utilization (attendance) and untreated caries among 3-6 year old Medicaid-enrolled children attending well-child visits (WCV) in primary care settings. The focus is on addressing factors (determinants) at the socio-ecological levels of the child's environment: provider (pediatrician and nurse practitioner), parent/caregiver and practice/organization levels. Eighteen practices will be randomized to 2 arms for provider training and practice/organization level study intervention changes: A) provider-level CSM training, provision of oral health facts to parents, a prescription and a list of dentists accepting Medicaid + practice-level EMR changes for documenting oral health; B) AAP training with no prescription or list of dentists accepting Medicaid and no changes to the EMR. Arm A parents/caregivers will receive oral health facts and prescription to take their child to the dentist and improve oral health behaviors in the home, while Arm B parent/caregivers will receive usual AAP-based care for oral health. Each arm will consist of 9 practices (n= 18), 33 to 34 providers (n= 67), and 512 parent/caregiver and child dyads (n= 1024). Each parent/caregiver and child dyads will be recruited at the first WCV and then followed for two consecutive WCVs (for a 24 month duration *or* until the subject has completed 3 well child visits). Each provider will complete the didactic and skills training prior to enrolling any parent/caregiver and will participate in the study for a total of 24 months duration (includes initial and booster didactic and skills training: 3 months, WCV#1: 3 months, WCV#2: 3 months, WCV#3: 3 months). Data will be collected prior to WCVs, at WCVs, and follow-up visits.

Immediately after randomization of practices, recruitment will be rolled-out, i.e. parent/caregivers will be recruited during a 3-month period in 6 practices at a time, with recruitment at all 18 practices expected to be completed in 9 months. This strategy will maximize staff time and ensure that EMR integration of OH is complete at practices randomized to Arm A, and that providers receive the didactic and skills training prior to their practice's rollout.

4.1 Study participants

Subjects will be pediatric providers (Pediatricians/Nurse Practitioners) and parent/caregivers and their 3-6 year old children from 18 primary care practices located in Ashtabula, Cuyahoga, Geauga, Lorain, Medina, and Portage counties in Ohio (see Table 4.1.1 below). One additional practice not included in the table, Premier Pediatrics, has also agreed to participate and will be included if needed to replace a practice. The majority of the practices are in Cuyahoga County (67%) and the remaining 33% are in surrounding counties in NE Ohio. All practices serve ≥ 20% Medicaid eligible children (9 practices with 20-40% and 9 practices with > 40% Medicaid-eligible children) and are from the Rainbow Care Connection (RCC). The study is offered to all pediatricians/nurse practitioners in the recruited practices and will be offered to all eligible caregivers and their children excluding those with serious medical or behavioral conditions which would preclude them from participating in the dental screening. All provider and parent participants meeting the eligibility criteria will be enrolled in the study upon signing the consent form.

Table 4.1.1 Practice Information

Confidential

4.2 Model and Design

Figure 4.2.1 illustrates the pathway through which the behavioral (provider-level) and implementation (practice-level) interventions are intended to result in improved receipt of dental care. The theory-driven interventions were developed using two frameworks: socio-ecological[51,52] and intervention mapping.[45] Thus, on a provider-level, pediatricians' and nurse practitioners' communication of OH facts + prescription along with list of dentists are intended to change parental caries illness perception (from disjointed inaccurate → chronic organized understanding) and are intended to change self-efficacy to seek dental care for their child. On a practice level, the integration of oral health and systematic documentation in EMR supports uniform data collection and enhances continuous quality improvement to facilitate providers' follow-up with the parent at the next WCV.

Study measures/evaluations (See Table 8.1.1) will utilize the RE-AIM framework[53,54] similar to a hybrid designed study.[55] Internal validity is assessed by effectiveness and implementation/fidelity; external validity is assessed by reach, adoption, and maintenance (See Figure 4.2.1). Child primary (i.e. dental attendance) and secondary (i.e. development of new caries, changes in oral hygiene, frequency of sweetened food and drinks, oral health-related quality of life, and dental costs) effectiveness outcomes will be mediated by changes in parent's illness perception and self-efficacy. Other external validity outcomes as a result of provider- and practice-level interventions via mediators (provider's OH knowledge and self-efficacy; practice's leadership completing the organization readiness, climate, and attitude questionnaire) will also influence the child outcomes. A literature search on the predictors of dental attendance identified moderating variables such as caregivers' sociodemographics,[56] health literacy[,57] social support[58,4] and child medical illness as a confounder.[59,60] Thus, all measures and evaluations collected for this study will follow the conceptual model.

For the purposes of dissemination of cRCT study results and the intervention components to all stakeholders, the study will utilize Roger's Diffusion of Innovation3 framework for evaluation and development of a toolkit. Specifically, process outcomes collected as part of implementing the cRCT together with additional structured interview data (to be collected from participating providers) on barriers/enablers will help in understanding the characteristics of the intervention and any changes that would need to be made for dissemination purposes.

Figure 4.2.1 Conceptual model describing the pathway mechanisms of the behavioral- (provider) and implementation- (practice) level interventions

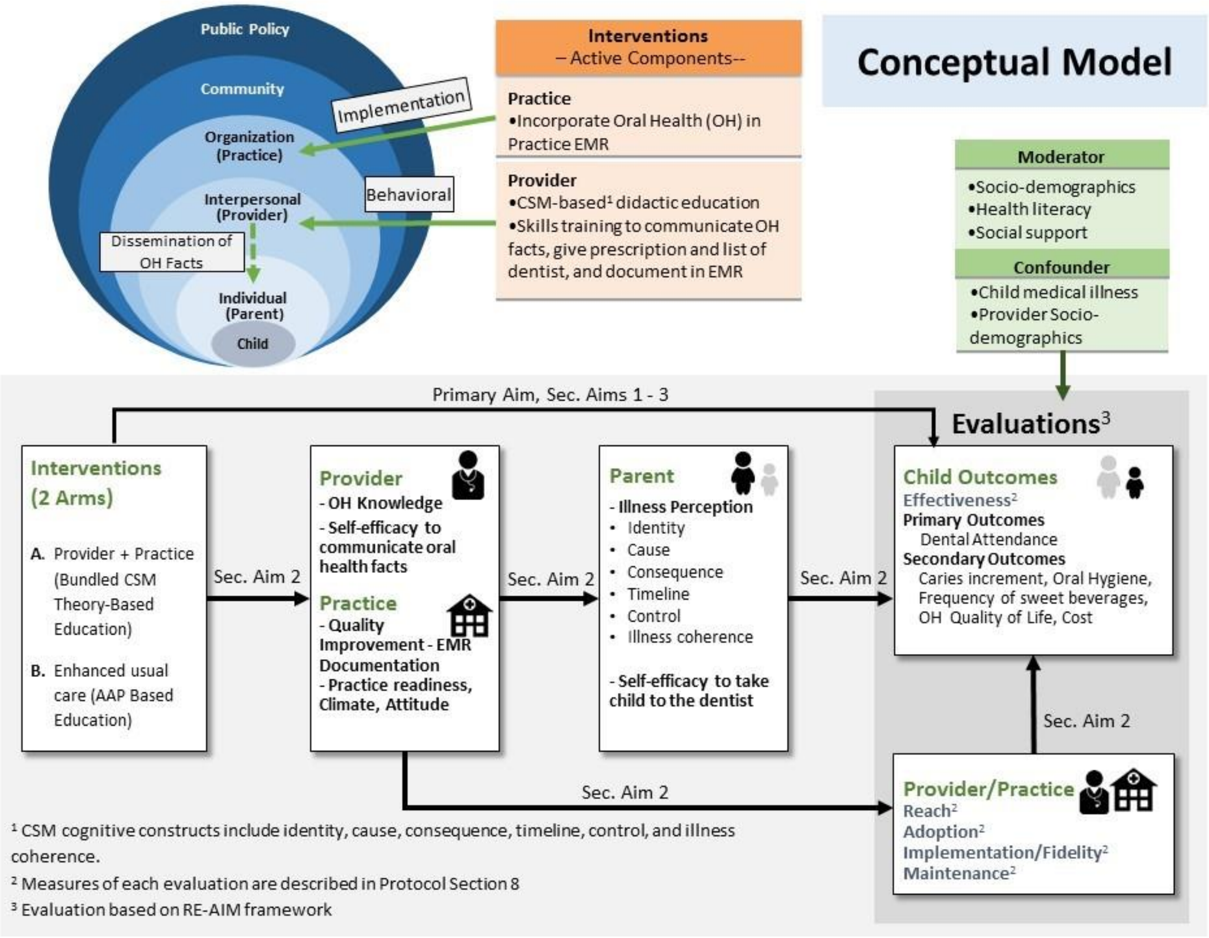

Interventions to be tested:

Arm A (N = 512): Practices will receive interventions at the provider and practice levels.

<u>Provider-level interventions will be</u>: Common-Sense Model of Self-Regulation (CSM) based education and skills training for the provider to teach him/her to communicate core oral health facts to parents, provide a prescription to take their child to the dentist together with a list of Medicaid-accepting dentists in the area, and document the oral health encounter in EMR.

<u>Practice-level interventions will be</u>: Enhancements to the EMR system to include oral health documentation (four questions) which will be implemented prior to enrolling any parent/caregiver and child participants into the study.

Arm B (N = 512): Enhanced usual care (AAP based) at the provider level, no practice level intervention.

Providers in these practices will receive American Academy of Pediatrics (AAP) based oral health education and follow the usual care for oral health assessment recommended by AAP guidelines. The AAP based education is unlike the CSM based intervention education (given to Arm A) in that it is not theory based to provide understanding of the chronicity of dental caries.

4.3 Design of Supplementary Objective 3.3

Qualitative data collection to evaluate dissemination strategies to translate trial results and resources to all stakeholders will also be completed. Specifically, we will use the final trial results, the process outcomes collected during the study, and additionally collect structured interview data from participating providers on implementation barriers/enablers to evaluate best strategies for dissemination of the oral health intervention. In addition to disseminating trial results, a tool kit will also be developed to translate the evidence based intervention among all primary care stakeholders.

The 63 participating providers will be asked to participate in a structured interview to assess the characteristics of the intervention and implementation barriers/enablers according to Roger's Diffusion of Innovation framework. These interviews will be collected from the providers after the completion of all parent/caregiver-child dyad WCV3 visits. A trained moderator will conduct the interviews (≈45 minutes) at the practices to assess the following: 1) relative advantage of the OH intervention; 2) compatibility of the OH intervention with the needs of the adopters; 3) Complexity of the OH intervention; 4) Trialability, i.e. implementing the OH intervention without a large investment; 5) observability, i.e if the results of the OH intervention are noticeable to parents and providers. The table below gives the list of questions that will be asked under each domain. All interviews will be audio-taped. Audio tapes from the interviews will be transcribed, and then coded in Atlas.ti, a software program which allows for the analysis of qualitative data. Analysis will be supervised by Drs. Nelson who has experience conducting qualitative work. A detailed summary of the ideas for implementation strategies and development of the tool kit will be produced. Interviews with providers will be conducted using zoom if in-person interviews are not possible due to the current COVID-19 outbreak.

Diffusion of Innovations Theory Characteristics	*Structured interview Questions for Providers*
(1) Relative advantage; the degree to which the Innovation is viewed as better than the previously available ideas or programs.	*Is the information in the didactic oral health (OH) curriculum you received as part of training better than the ones available on professional websites (ADA, AAP, and AAPD)?*<br>a. *Was anything new presented?*<br>b. *Did you learn anything?*<br>*Can the curriculum with the OH facts be delivered successfully to parents? Should the training be made available from experts?*<br>a. *Has skills training been helpful?*<br>b. *Does it give confidence in delivery?*<br>c. *Is in-person training necessary?*
(2) Compatibility: the degree to which the innovation is consistent with the values, experiences, and needs of potential adopters.	*Are the OH activities in primary care compatible with physicians/NP's goal of quality of care delivery?*<br>a. *What are the enablers?*<br>b. *What are the barriers?*
(3) Complexity: How difficult the innovation is to understand or how complex it is to use.	*Are the OH activities simple to be followed in practice settings?*<br>a. *What could be made simpler?*<br>b. *What works and what does not?*

(4) Trialability: the degree to which the innovation can be experimented with on a limited basis without a large investment.	*Has the implementation of OH activities been cost-effective?*
(5) Observability: the degree to which the results of an innovation are visible to others.	*Do you think the OH activities have helped your parents to take their child to the dentist and also helped with Quality Improvement in your practice?*

5 STUDY ENROLLMENT AND WITHDRAWAL

5.1 Subject Inclusion Criteria

In order to be eligible to participate in this study, all of the following criteria must be met:

Practices:

- Use Electronic Medical Record (EMR)
- Have ≥ 20% of pediatric patients covered by Medicaid

Providers:

- Pediatrician or Nurse Practitioner with a minimum of 2 patient-care days per week
- Provide signed and dated consent form

Parents or caregivers:

- Legal guardianship of Medicaid-enrolled children aged 3-6 years attending well-child visit (WCV)
- Must be ≥ 18 years
- Speak English or Spanish
- Provide signed and dated consent form
- Planning to stay in the immediate area (both parent/caregiver and child) for at least two years

Child:

- Ages 3-6 years
- Enrolled in Medicaid

5.2 Subject Exclusion Criteria

An individual who meets any of the following criteria will be excluded from participation in this study:

Child:

- Presence of any serious medical or behavioral condition (e.g. cerebral palsy, autism) that precludes participation in the dental screening

5.3 Strategies for Recruitment and Retention

Practices:

During the pilot study phase, Medical Directors from the 26 RCC practices were invited to participate in an informational webinar led by the PI and a Co-Investigator (Medical Director of the RCC) to learn more about the opportunity for their practice to participate in the study. The PI, Project Manager and Study Coordinator then met with interested providers at practices which met the inclusion criteria. The 18 practices which met the inclusion criteria have provided letters of support indicating their commitment to participation. One practice will serve as a backup practice and has also given a letter of support.

Retention strategies for the practices will include regular contact between the study staff and practice staff to ensure any issues which may arise are addressed immediately, and tailoring study logistics based on practice flow as learned from our two pilot practices.

Practices also receive compensation for facility use for the trainings, for research staff being present at the practices to complete study visits, and for the structured interviews and debriefing sessions.

Providers:

During the pilot study phase, all pediatricians and nurse practitioners of the 18 practices were invited to attend the informational meetings which took place at their individual practices. The PI, Project Manager and Study Coordinator briefed the providers regarding the study goals and logistics. A total of 67 providers from the 18 practices to be randomized who met the inclusion criteria will participate in the main trial. An additional information session for providers will be held at each practice prior to the start of the main trial. At this time, informed consent will be obtained from each pediatrician/nurse practitioner participant.

Retention strategies for providers include Continuing Medical Education (CME) credits for didactic OH education and Maintenance of Certification (MOC) credits for quality improvement initiatives. Twenty five (25) MOC credits have been approved by the American Academy of Pediatrics (AAP) for physicians participating in this dental project. The study staff will maintain regular contact with providers to address any concern or issue that may arise.

CME credits will also be available for providers upon completion of briefing seminar.

Parent/caregivers:

All parents of 3-6 year-old Medicaid-enrolled children attending a WCV in the selected practices during the recruitment period will be invited to participate in the study. Based on the data from the 18 practices, we expect 7,421 Medicaid-enrolled children aged 3-6 years to have a WCV at the pediatric offices per year. In our pilot study, the participation rate was 95%, hence similar strategies will be implemented in the main trial.

A team consisting of a research assistant and dental hygienist will be assigned to one practice at a time to be on-site daily for recruitment of caregiver-child dyads at larger practices. A floating team will recruit at multiple smaller practices simultaneously. The available sample pool for the 3-month recruitment period is 1,855 (25% of 7,421). We anticipate that 5% will be ineligible based on inclusion and exclusion criteria, thus reducing the potential sample to 1,762 (95% of 1,855). Based on our experiences recruiting and enrolling parent/caregivers in 2 pilot practices, we expect a participation rate of 95% (of 1,762), giving us an available potential sample size of 1,674 per 6 practices enrolled. For the total of 18

practices, we can expect a potential sample size of 5,022 from whom we can recruit the target sample size of 1024 participants (512 per arm for 2 arms) required to test the hypothesis of the study. Therefore, our goal of recruiting 56 to 57 participants per practice given the potentially large population pool of each practice is an achievable approach to meet the main trial sample size. The recruitment is expected to be completed within a 9-month time frame.

The recruitment and enrollment strategies proved to be effective in the pilot study will be used for the main trial as follows:

- When making appointment reminder calls, practice staff will give a brief overview of the study (using a recruitment script) and ask caregivers to come early to fill out the consent forms and questionnaires.
- Tailored to the practice's or provider's preference, the medical assistant may approach caregivers first, before study staff explain and obtain consent. An alternative approach is that study staff will approach caregivers in the waiting room.
- All 3 questionnaires, i.e. Illness Perception Questionnaire-Revised for Dental (IPQ-RD), Parent Questionnaire, and Parent Exit Questionnaire, can be completed on paper or tablet according to the caregiver's preference.
- At each WCV, study staff will give caregivers an incentive (i.e. cash or gift card) and a small gift for their child for completing the study visit questionnaires.

Retention strategies for parent/caregivers, lessons learned from the pilot study and best practices identified in the literature will be followed as below:

- Parents/caregivers will be given promotional items (i.e. pens, magnets) with the study logo and contact phone number at recruitment.
- Alternate contact information for family/friends that may be able to reach the participant if primary contact information becomes invalid will be obtained at recruitment.
- Annual birthday/holiday cards will be sent to parent and child participants to maintain contact.
- Newsletters will be sent twice a year with updates on the study's progress (i.e. recruitment), and a reminder to update their contact information (by phone or mail).
- Study staff will contact parents/caregivers during follow-up to remind them to schedule and attend their annual WCV.
- Assistance from practice staff will also be solicited for hard to reach/contact participants.

5.4 Treatment Assignment Procedures

5.4.1 Randomization Procedures

Eighteen practices (comprising the clusters) will be included in the study. Practices will be randomized to one of the two arms using a restricted randomization scheme with the constraint that 9 practices will be assigned to each arm. This approach will involve the computation of a balance score (for each candidate randomization) based on marginal differences in three key practice-level variables: % Medicaid-enrolled patients [20% to 40% and >40%], ratio of patients to providers, county [Cuyahoga vs. other]). We use the

first generated (constrained) randomization that provides good balance according to a pre-selected balance criterion; it may be reasonable to require perfect marginal balance, if (as we expect) there are a reasonably large number of randomizations that yield this.

5.4.2 Masking Procedures

Parent/caregiver participants will be blinded to the intervention arms of the main trial. Furthermore, parent/caregiver participants will not be aware of what is involved in the study procedures being followed at other clinics. Study staff conducting dental screenings will also be blinded.

5.5 Subject Withdrawal

5.5.1 Reasons for Withdrawal

Participants are free to withdraw from participation in the study at any time upon request, as outlined in the consent form documents.

Participants who drop out (or withdraw) from the study by their own choice will be considered dropouts/withdrawals. Participants who are discontinued from the study by one of the study personnel will be also be considered a dropout/withdrawal.

An investigator may terminate a study subject’s participation in the study if:

- Any medical condition or situation occurs such that continued participation in the study would not be in the best interest of the participant.
- The subject meets an exclusion criterion (either newly developed or not previously recognized) that precludes further study participation.

5.5.2 Handling of Subject Withdrawals or Subject Discontinuation of Study Intervention

All reasons for dropout/withdrawal or discontinuation of the study intervention will be documented in the tracking database and reviewed by the study team and reported to the NIDCR Medical Monitor and DSMB. For gathering information on discontinuation, study staff will be contacting subjects at several time points. Study staff will contact parent/caregivers within 2 weeks of WCVs #1 and #2 to collect B1 questionnaires not previously returned, 6 months after WCVs #1 and #2 to complete B2 questionnaires, and 3 months and 2 weeks before WCVs #2 and #3 to remind caregivers to schedule/attend WCV.

In the event of a subject telling study staff that he/she is dropping out of the study, the study staff should gather the following information as much as the participant allows.

- Record the reason for dropping out of the study.
- Record any adverse event reported by the subject.
- Complete the appropriate questionnaire nearest the drop out time point.

If it is determined by study staff that a participant should be discontinued from the study, the decision to withdraw a participant must be discussed and confirmed by the PI.

Caregiver/child dyads that withdraw during the baseline (T_0) data collection window will be replaced by recruitment of additional parent/child dyads to reach enrollment goals.

Caregiver/child dyads that withdraw or that are lost to follow-up any time after the baseline data collection window will not be replaced, but data collected up to the point of withdrawal will be used for analysis.

Providers that withdraw after receiving the study intervention will not be replaced. See Section

7.1.4.

5.6 Premature Termination or Suspension of Study

This study may be suspended or prematurely terminated if there is sufficient reasonable cause. Written notification, documenting the reason for study suspension or termination, will be provided by the suspending or terminating party to the Principal Investigator (PI), Medical Monitor/DSMB, or NIDCR. If the study is prematurely terminated or suspended, the principal investigator will promptly inform the IRB and will provide the reason(s) for the termination or suspension.

Circumstances that may warrant termination include, but are not limited to:

- Determination of unexpected, significant, or unacceptable risk to subjects.
- Insufficient adherence to protocol requirements.
- Data that are not sufficiently complete and/or evaluable.
- Determination of futility.

6 STUDY INTERVENTION

Refer to **Appendix B** for more details on section 6 in the Fidelity Monitoring Plan.

6.1 Study Behavioral or Social Intervention(s) Description

Complete descriptions of the interventions are given in the fidelity monitoring plan (Appendix B).

The interventions for this study are on two levels as follows: *Provider:* Common-Sense Model of Self-Regulation (CSM) based education and skills training for the pediatrician/nurse practitioner to understand chronicity of caries, and communicate the core OH facts to the parent, provide a prescription to take the child to the dentist together with a list of area dentists accepting Medicaid, and document OH in EMR. *Practice:* enhancements to the EMR system to include OH status will be implemented prior to enrolling any parent into the study. This will promote adoption of systematic EMR documentation of OH status that can facilitate follow-up with parent at subsequent WCVs.

Table 6.1.1: Theoretical perspectives for behavioral interventions at each level

Socio-ecological levels	Change objectives	Theoretical methods (theory)	Practical application and delivery of interventions
**Individual (parent/caregiver)**	• Learn chronicity of caries and seriousness regarding primary teeth<br>• Behavioral capability / awareness for seeking dental care<br>• Skills & self-efficacy for taking child to dentist	• Information processing and transfer (***CSM***)<br>• Self-management (***CSM, SCT***)<br>• Active learning with images (***CSM, SCT***)<br>• Reinforcement (***SCT***)	• OH assessment and facts from primary care doctors<br>• Educational materials with illustrations<br>• List of dentists
**Interpersonal (provider)**	• Behavioral capability / knowledge of core OH facts<br>• Skills & self-efficacy to communicate core OH facts<br>• Refer to dentist for dental care	• Information processing and transfer ***(CSM)***<br>• Active learning ***(SCT)***<br>• Persuasion ***(communication)***<br>• Reinforcement ***(SCT)***	• Brief lectures through seminars, lunch and learn, webinars etc.<br>• Skills and communication training to impart one-on-one OH facts to parent/caregivers<br>• Referral to dentist together with list of Medicaid accepting dentists

**Organization (practice)**	• Awareness of OH in practices<br>• Integrate OH assessment in EMR<br>• Systematically document OH for quality improvement and monitoring	• Information transfer ***(CSM)***<br>• Identifying practice attributes and champion/s ***(Diffusion of Innovation theory)***<br>• Active learning ***(SCT)***<br>• Feedback ***(SCT)***<br>• Creating OH protocols ***(SCT)***	• EMR enhancement and prompts for OH documentation<br>• Skills training of practice providers and staff to document<br>• Standardized OH protocols<br>• Newsletters to communicate progress in practices

CSM – Common Sense Model of Self-Regulation, SCT – Social Cognitive Theory

6.2 Administration of Intervention

Arm A

The provider-level intervention consists of CSM theory based didactic education and skills training for providers to learn about the chronicity of caries, to communicate core oral health facts to parent/caregivers at the well-child visit (WCV), to provide a prescription to take their child to the dentist together with a list of area Medicaid-accepting dentists, and to document OH in EMR. By enhancing dental knowledge and skills of the pediatric providers through the CSM-based education, interactive practice sessions with standardized patients, and training in EMR documentation, parents/caregivers will receive the core OH facts along with coping and action supports (e.g., prescription to dentist, list of Medicaid accepting dental providers) from the child's provider. Providers will receive didactic education with pre- and post-tests and interactive skills training with standardized patients (SPs). Providers will also be given materials (Appendix D) to help them implement the intervention including: training manual that includes a didactic section on oral health and skills section on communication (more specifics on the training manual will be detailed in the MOP), pocket card (with core oral health facts listed), and flip chart (to use during oral health communication to caregivers). The training will be delivered to providers in two 45-minute sessions (didactic education first and then skills training) at their individual practice prior to study enrollment of caregiver and child participants at WCV #1. There will be a booster education session before WCV #2. The final WCV #3 will serve as the maintenance visit.

Arm A will also have practice-level intervention that includes incorporation of four oral health questions into the practice EMR. As a result of this practice-level intervention, Arm A providers will receive EMR training to document the oral health encounter. The EMR training will occur simultaneously with the didactic and skills training sessions prior to WCV #1 and the booster session before WCV #2. Providers will receive an EMR training manual for documentation (more specifics on the EMR training manual will be detailed in the MOP; see Appendix D for EMR screenshots). While the majority of University Hospitals based practices have oral health questions incorporated in their EMR, the OH questions are hidden and will be available only to providers with appropriate training in practices in study Arm A. The other non-UH based practices randomized to study Arm A will have the OH questions incorporated prior to enrollment of caregiver and child participants.

Parents/caregivers of the providers and practices in Arm A are the recipients of the provider and practice level interventions. Parent/caregivers will receive CSM-based oral health facts, prescription to go to the dentist together with a list of Medicaid-accepting dentist.

Arm B

Providers will receive the standard AAP based didactic education in a 45-minute session prior to WCV #1and a booster session prior to WCV #2. Arm B providers will not receive any CSM-based education, skills training or the practice-level intervention.

Parents/caregivers in this arm will receive the usual care for oral health as followed by the providers and practices currently.

6.3 Procedures for Training Interventionists and Monitoring Intervention Fidelity

Refer to **Appendix B** for the Fidelity Monitoring Plan for the study.

All study staff will receive Human Subject Protection, Good Clinical Practice (GCP), protocol, and MOP training.

6.3.1 Procedures for Training Interventionists

Provider training (Arm A) will be conducted by experts on the curriculum team: Drs. Nelson (who has more than 20 years of experience as an oral health researcher), Ferretti (who has spent more than 20 years teaching residents as chief of pediatric dentistry), and Lord (a communication specialist with more than 10 years of teaching experience in the area of primary care delivery). The provider will need to complete three components satisfactorily to be considered as trained: (1) didactic training (assessed through pre and post test); (2) skills training (assessed through standardized communication checklist, and self-efficacy pre and post test); (3) observation of the first OH encounter of the provider by study staff (assessed through the standardized communication checklist. A score card will be given that outlines the established target goals which are elaborated in detail in the Fidelity Monitoring Plan in Appendix B).

Training materials, including scripts and narrated slides, will ensure that the curricula are delivered in the same way for each group of clinicians to ensure fidelity of the training delivery. A training manual (for Arm A) more specifics will be detailed in the MOP), EMR training (only Arm A) and flip chart and pocket card (Arm A) have been developed to provide information as needed (Appendix D, also includes EMR screen shot). Additional booster training for providers will be conducted, if necessary, to address deviations in protocol.

6.3.2 Procedures for Monitoring Intervention Fidelity

Arm A

Providers' implementation of the provider-level intervention will be assessed at all 3 WCVs. Two - three of each provider's OH encounters with parent/caregiver will be observed or audiotaped and then

evaluated by the study staff using a standardized communication skills checklist (Appendix E) at WCV #1 and WCV#2. The results of the observation/audiotaping will be given to the provider as a scorecard to provide feedback regarding whether the provider met the established target goals which are elaborated in detail in the Fidelity Monitoring Plan (Appendix B). All enrolled caregivers will complete an exit questionnaire (Appendix E) after the OH encounter to assess whether the provider communicated the OH facts and gave them the prescription and list of dentists. So, *Provider-level fidelity measures* to assess whether providers communicated the core OH facts to caregivers and delivered the prescription to go to the dentist and list of area Medicaid-accepting dentists include: 1) standardized communication skills checklist completed by study staff and 2) parent exit questionnaire (Appendix E).

Providers' implementation of the practice-level intervention will be assessed at all 3 WCVs. Study staff will complete monthly EMR audits (at all 3 WCVs) of all enrolled patients' EMR chart to assess whether providers' OH documentation was complete.

The *practice-level fidelity measure* to assess adoption of the intervention is the EMR Audit of Practice form completed by study staff (Appendix E).

Arm B

Since Arm B does not receive the interventions, monitoring procedures outlined below will be used to monitor baseline rate and check for contaminations between study arms.

Two - three of each provider's OH encounters will be randomly selected for observation/audiotaping and evaluation by study staff using the same standardized communication skills checklist used in Arm A. Scorecards will not be given to Arm B providers, but evaluation with the checklist will serve to document the communication of OH facts due to enhanced usual care.

Study staff will complete monthly EMR audits using the EMR Audits of Practice form (at all 3 WCVs) for 20% of enrolled patients' EMR chart to assess whether any oral health documentation was completed despite not receiving EMR training. (Appendix E).

6.4 Assessment of Provider Compliance with Study Intervention

Arm A

Compliance with the provider-level intervention: The study staff's direct observation/audiotape of provider encounters will provide an assessment of whether providers communicated all 6 core OH facts and gave caregivers the prescription to go to the dentist along with list of area Medicaid-accepting dentists. It will also be used to assess whether the provider initiated the topic of the child's dental care with the caregiver and used appropriate communication skills. The exit questionnaire completed by parent/caregivers will assess whether their pediatrician/nurse practitioner communicated OH facts and gave them the prescription to go to the dentist and list of dentists (i.e. whether the caregiver received and understood the main points of the provider's oral health communication and the prescription they were being given). Compliance will also be verified with the numbered prescriptions and list of dentists to determine the total number given to each provider's enrolled caregivers.

Compliance with the practice-level intervention: The study staff will conduct EMR audits of all enrolled patients to determine whether the OH documentation was complete, i.e. provider documented whether or not they did the following during the OH encounter: 1) examined child's teeth for white or brown spots, 2) asked whether child had a dental visit in the past 12 months, 3) communicated core OH facts, and 4) gave caregiver a prescription to take their child to the dentist along with a list of Medicaid-accepting dentists in the area.

Arm B

Compliance with the provider-level intervention: Study staff evaluation of providers' OH encounters and parent exit questionnaires completed by a random 20% selection of each provider's enrolled patients will be used to assess whether Arm B providers communicated OH facts to identify possible contamination bias in delivery of the provider-level intervention (i.e. provider is giving caregivers provider-level intervention materials not associated with their assigned arm).

Study staff will perform EMR audits on a random 20% selection of each provider's enrolled patients to identify possible contamination bias in delivery of the practice-level intervention (i.e. providers are documenting patients' OH without having received the practice-level intervention).

7 STUDY SCHEDULE

Prior to any of the following actions, practices will be randomized and EMR changes will be finalized.

Refer to **Appendix A** for the Schedule of Events for providers and parents/caregivers.

7. 1 Provider

7.1.1 Baseline Visit: WCV #1

Before WCV #1 (T_{-1}: Day -30 to -1)

- Review the written consent form with the provider; study staff signs the consent form acknowledging that informed consent was reviewed and provider signs acknowledging consent was obtained (Appendix C: Consent forms).
- Provide OH didactic education and skills training for pediatricians/nurse practitioners to communicate core OH facts (importance of baby teeth, chronicity of caries, dental visits) to parents/caregivers (Appendix D: Provider training) or AAP based didactic training, based on study arm.
- Administer pre- and post-tests to pediatricians/nurse practitioners before and after the OH didactic education session (Appendix E: Questionnaires & forms).

At WCV #1 (T_0: Day 0)

- Provider delivers oral health facts, based on study arm.
- Provider documents OH in EMR, based on study arm.

7.1.2 Follow-up Visit: WCV #2

<u>Before WCV #2 (T_2: Day 364 ± 90)</u>

- Provide OH didactic education booster session for pediatricians/nurse practitioners.

<u>At WCV #2 (T_3: Day 365 ± 90)</u>

- Provider delivers oral health facts, based on study arm.
- Provider documents OH in EMR, based on study arm (Appendix D: Provider training)

7.1.3 Final Visit: WCV #3

<u>At WCV #3 (T_6: Day 730 ± 90)</u>

- Provider delivers oral health facts, based on study arm.
- Provider documents OH in EMR, based on study arm (Appendix D: Provider training).

<u>After WCV#3 (Day 731 +)</u>

- Revised consent form is reviewed with the provider; study staff signs the consent form acknowledging that informed consent was reviewed and provider signs acknowledging consent was obtained.
- Provider completes structured interview conducted by a trained moderator. The interviews will be conducted at the pediatric practices at a time convenient for the provider. Interviews will be conducted using zoom if in-person interviews are not possible due to the current COVID-19 outbreak. The interviews will contain questions to assess:
    - Relative advantage of the OH intervention
    - Compatibility of the OH intervention with the needs of the adopters
    - Complexity of the OH intervention
    - Trialability
    - Observability
- Provider attends debriefing session held at the practice.

7.1.4 Provider Withdrawal Visit

The following procedures will be followed for providers who choose to withdraw early or whose participation is terminated by the PI:

- Personal contact (by phone or in-person) by study staff will be made to confirm withdrawal.

- Debrief about the interventions.
- If a provider withdraws from the study and their patients are participants who see another provider at that practice, visits and data collection will continue. If the patient also withdraws by changing practices *to a non-participating practice*, visits and data collection will end with these participants being considered lost to follow-up. Data collected up to the point of withdrawal will be used for analysis.

7.2 Parent/Caregiver and Child

7.2.1 Baseline Visit: WCV #1

At WCV #1 (T_0: Day 0)

Before Provider Encounter

- Study Staff will recruit 1024 parent/caregivers and their children from the 18 practices (approximately 56-57 participants/practice) using an in-person approach. Parents/caregivers attending well-child visits with 3-6 year old children will be approached for participation.
  - In the waiting area/exam room of the pediatric practice, study staff will review inclusion/exclusion criteria as outlined on the screening survey to determine parent/caregiver and child eligibility (Refer to Sections 5.1, 5.2).
  - Review the written consent form with the potential participant; study staff signs the consent form acknowledging that informed consent was reviewed and caregiver signs acknowledging consent was obtained (Appendix C: Consent forms).
  - In the waiting area/exam room of the pediatric practice, study staff will obtain contact information.
  - In the exam room, caregivers will complete the following self-administered Baseline questionnaires: A1: Illness Perception Questionnaire-Revised for Dental (IPQ-RD) and A2: Parent Questionnaire (Appendix E: Questionnaires & forms).
- In the exam room, dental hygienist will perform child dental screening examination and study staff will record results of the dental screening examination. Results will not be available to the provider, unless the hygienist finds that the child has urgent needs that require immediate attention. (Appendix E: Questionnaires & forms).

During Provider Encounter in the Exam Room

- Caregivers attending WCV’s will receive OH information, prescription to take the child to the dentist + list of local Medicaid-accepting dentists from provider, based on study arm.
- Caregivers attending WCV’s may have study staff audiotape or directly observe the provider encounter to evaluate provider’s delivery of OH communication (for a sample of visits).

After Provider Encounter in the Exam Room/Waiting Area

- Caregivers will provide feedback about the OH information given to them during the medical visit with a short self-administered exit questionnaire (A3: [Appendix E: Questionnaires & forms]).
- Caregivers will be given the B1: Follow-up IPQ-RD (Appendix E: Questionnaires & forms) to be completed and returned (in postage paid envelope or in person, based on participant preference).

<u>After WCV #1 (T_1)</u>

- **B1 (Day 0 ± 120)**: Caregivers will receive telephone calls and text messages from study staff to remind them to complete and return the B1: Follow-up IPQ-RD if it has not already been returned.
- **B2 (Day 180 + 60)**: Caregivers will receive telephone calls and text messages from study staff 6 months after the WCV to complete the B2: Dental Attendance & Cost Questionnaire (Appendix E: Questionnaires & forms).

7.2.2 Follow-up Visit: WCV #2

<u>At WCV #2 (T_3: Day 365 ± 90)</u>

Before Provider Encounter in the Exam Room

- If a different parent/caregiver brings the child to the well child visit and they are the child's legal guardian, consent will be obtained from that parent/caregiver and they will complete all components of the visit.
- Parent/caregivers will complete the following self-administered questionnaires: A1: Illness Perception Questionnaire-Revised for Dental (IPQ-RD) and A2: Parent Questionnaire.
- Dental hygienist will perform child dental screening examination and study staff will record results of the dental screening examination. Results will not be available to the provider unless the hygienist finds that the child has urgent needs that require immediate attention.

During Provider Encounter in the Exam Room

- Caregivers attending WCV's will receive OH information, prescription to take the child to the dentist + list of local Medicaid-accepting dentists from provider, based on study arm.
- Caregivers attending WCV's may have study staff audiotape or directly observe the provider encounter to evaluate the provider's delivery of OH communication (for a sample of visits).

After Provider Encounter in the Exam Room/Waiting Area

- Caregivers will provide feedback about the OH information given to them during the medical visit using a short self-administered exit questionnaire (A3).

- Parents will be given the B1: Follow-up IPQ-RD to be completed and returned within 2 weeks (in postage paid envelope or in person, based on participant preference).

<u>After WCV #2 (T_4):</u>

- **B1 (Day 365 ± 120)**: Caregivers will receive telephone calls and text messages from study staff to remind them to complete and return the B1: Follow-up IPQ-RD if it has not already been returned.
- **B2 (Day 545 ± 90**): Caregivers will receive telephone calls and text messages from study staff 6 months after the WCV to complete the B2: Dental Attendance & Cost Questionnaire (Appendix E: Questionnaires & forms).

7.2.3 Final Visit: WCV #3

<u>At WCV #3 (T_6: Day 730 ± 90)</u>

Before Provider Encounter in the Exam Room

- If a parent/caregiver will be asked to complete the COVID-19 questionnaire, they will be re-consented with the updated consent form (12/7/20).
- Parent/caregivers will complete the following self-administered questionnaires: A1: Illness Perception Questionnaire-Revised for Dental (IPQ-RD) and A2: Parent Questionnaire, COVID-19 Dental Visit Questionnaire (if applicable)
- Dental hygienist will perform child dental screening examination and study staff will record results of the dental screening examination. Results will not be available to the provider unless the hygienist finds that the child has urgent needs that requires immediate attention

During Provider Encounter in the Exam Room

- Caregivers attending WCV's will receive OH information, prescription to take the child to the dentist + list of local Medicaid-accepting dentists, based on study arm.
- Caregivers attending WCV's may have study staff audiotape or directly observe the provider encounter to evaluate the provider's delivery of OH communication (for a sample of visits).

After Provider Encounter in the Exam Room/Waiting Area

- Caregivers will provide feedback about the OH information given to them during the medical visit using a short self-administered exit questionnaire (A3). Actively enrolled parents will also complete a short (7 item) COVID-19 Questionnaire which asks if they have altered their behavior related to taking their child to the dentist due to the COVID19 pandemic.

7.2.4 Parent/Caregiver and Child Withdrawal Visit

The following procedures will be followed for participants who choose to withdraw early or whose participation is terminated by the PI:

- Personal contact (by phone or in-person) by study staff will be made to confirm withdrawal.
- Record any adverse event reported by participant.
- Debrief about the interventions.
- Obtain responses to the study questionnaires (if applicable)
- If a parent/caregiver and child withdraws or changes to a practice not participating, visits and data collection will end with these participants being considered lost to follow-up. Data collected up to the point of withdrawal will be used for analysis.

7.2.5 Parent/Caregiver and Child Unscheduled/*Missed* Visit

Unscheduled visits for parent/caregiver and child may occur if an enrolled child could not complete the oral health portion during the WCV. But, this is unlikely to happen due to the short amount time required for the oral health activities. Another possibility would be if a child is scheduled by the practice for a well child visit much sooner or later than the visit window. In these cases, study data would be collected per guidelines outlined in the MOP, but the time length between visits will be accounted for in the analysis stage.

Other instances of unscheduled visits at follow-up, i.e.WCV2 or WCV3, may occur if a well child visit is missed by project staff (for example, a child comes in for a sick visit near the time a well child visit is due and the provider completes the well visit also) or another non-participating provider at the practice is scheduled to see the child for a well child visit. In these cases, the ICDAS, A2, A3 and B1 questionnaires will be completed when possible, either in person, by mail or through a link sent via text or e-mail to the parent/caregiver. The B2 follow-up will be completed as planned. In extreme situations, if we have missed the WCV2 or WCV3 and the child is scheduled to come in to see the enrolled provider for follow-up for a problematic issue within 3 months of the missed well child visit, we will arrange to complete the study visit at the follow-up visit. This determination will be made on a case-by-case basis. Arrangements will be made to have the parent/caregiver bring the child to the Dental Research Building for completion of the ICDAS dental screening. Also, due to scheduling irregularities in the part of the parent/caregiver, every child may not be scheduled for an annual WCV. In cases such as this, the parent/caregiver-child dyad may remain enrolled in the study and be seen for completion of 3 consecutive WCV's regardless of their timing. The time length between visits will be accounted for in the analysis stage.

COVID-19 Visits:

Visits missed due to the COVID-19 pandemic will follow the protocol outlined above for unscheduled/missed visits.

8 STUDY PROCEDURES / EVALUATIONS

8.1 Study Procedures/Evaluations

The following is a summary of the study procedures and evaluations. Study procedures include interventions, and procedures performed to assess the primary outcome, secondary outcomes, mediators, moderators and confounders. Measures or interventions will occur at the socioecological levels of child, parent/caregiver, provider, and practice. There will also be implementation evaluation at each of these levels except the parent/caregiver. See Table 8.1.1 for more details on the measures, sources, and timeline. According to the timeline of study procedures and evaluations, WCV #1: Baseline Visit = T_0; WCV #2: Follow-up Visit = T_3; and WCV #3: Final Study Visit = T_6.

Table 8.1.1 Summary of measures, sources, and timeline

Socioecological levels	Implementation Evaluation	Measures	Score	Source	Timeline
Child	<u>Reach</u><br># recruited<br>% attrition<br>Participant characteristics (socio-demographics)	<u>Effectiveness</u><br>**Primary outcome:** Dental attendance<br><br>**Secondary outcomes:** New caries<br>Oral hygiene<br><br>Frequency of sweet food & drinks<br><br>OH-related quality of life<br><br>Cost	% frequency<br><br># of decayed, filled teeth (dft) % freq. tooth brushing<br>% freq. sweet food & drinks<br><br>ECOHIS overall score<br><br>Direct dental costs<br>Non-dental direct & dental indirect costs	ICDAS; [61]<br>Medicaid claims<br><br>ICDAS [61] NHANES III (CDC)[62]<br><br>Food Frequency Qx, NHANES 2003-2004[2]; YRBS 2017[63]<br>[50]<br><br>Medicaid claims<br>Annotated Cost Qx[64]	T_0, T_1, T_3, T_4, T_6, T_7<br><br>T_0, T_3, T_6<br>T_0, T_3<br><br>T_1, T_4, T_7
		**Confounder:**<br>Child medical illness	Overall score	[60]	T_0, T_3, T_6
Parent/ caregiver		**Mediators:** IPQ-RD<br><br>Self-efficacy<br><br>**Moderators:**<br>Socio-demographics<br>Health literacy Social support	Overall & mean construct scores<br>Overall score<br><br>% categories<br>% mean scores	Nelson, 2016[65]<br><br>NHANES III (CDC)[62]<br>[28]<br>[66]	T_0, T_1, T_3, T_4, T_6<br><br>T_0, T_3, T_6
Provider	<u>Implementation/ Fidelity</u><br>Observation/ audiotape<br>Parent Exit Qx<br><br><u>Adoption & Maintenance</u><br>% prescriptions + list of dentists given	**Mediators:**<br>OH knowledge<br><br>Self-efficacy<br><br>**Confounder:**<br>Socio-demographics	% mean scores<br><br>% categories	Open Wide (NMCOHRC)<br>PACT (AAP)<br>[67-68]<br><br>NHANES III (CDC)	T_{-1}, T_2<br><br>T_{-1}

Practice	Adoption & Maintenance<br>% complete EMR documentation Practice Qx	**Mediators:**<br>EMR Documentation	% mean scores	69	T_4 and T_6
		Practice readiness, climate, and attitude	% mean scores	70	T_{-1}, T_2,

T_0 = WCV #1: Baseline Visit (1st intervention)
T_3 = WCV #2: Follow-up Visit (Booster intervention);
T_6 = WCV #3: Final Visit (Follow-up)
T_{-1} = Before WCV #1; T_0 = At WCV #1; T_1 = After WCV #1
T_2 = Before WCV #2; T_3 = At WCV #2; T_4 = After WCV #2 T_5 = Before WCV #3; T_6 = At WCV #3; T_7 = After WCV #3

8.1.1 Child-level

The primary (i.e. dental attendance) and secondary outcomes (i.e. new decay, oral hygiene, frequency of sweet food and drinks, oral health-related quality of life, and dental-related costs) are also implementation measures of effectiveness. Child medical illness is a confounder.

Child dental attendance (i.e. receipt of dental care) will be assessed through:

- Clinical dental screenings will be performed at all 3 WCVs (T_0, T_3, and T_6) by a trained and calibrated dental hygienist according to the International Caries Detection and Assessment System (ICDAS). The screenings will be used to identify a change in oral health status between WCVs #1 and #2 (T_0 and T_3) and WCVs #2 and #3 (T_3 and T_6) which is defined as a restoration (ICDAS ≥ 3) or extraction of ≥ 1 tooth previously identified with an active ICDAS lesion code ≥ 3 at T_0 or T_3 that would not be exfoliated naturally based on child's age. See ICDAS Assessment form (Appendix E). More details on the screening and decision algorithm will be outlined in the MOP Section 5.
- B2: Dental Attendance & Cost Questionnaire: Study staff will contact caregivers 6 months after WCVs #1 and #2 (T_1 and T_4) to ask whether their child had a dental visit or if they made a dental appointment for their child since the previous WCV (Appendix E).
- Medicaid claims files: Data will be abstracted by study staff to verify child's receipt of dental care after each WCV (T_1, T_4, and T_7) and entered on the Medicaid Claims Abstraction form. Current Dental Terminology (CDT) codes used for billing will be used to determine if the child received preventive or restorative procedures in the past 12 months. Dental emergency department (ED) visits will be identified by linking diagnosis codes (Appendix E) to ED claims using the International Classification of Diseases, Tenth Revision, Clinical Modification (ICD-10-CM). See Medicaid Claims Abstraction form (Appendix E) for preventive and restorative CDT codes and ICD-10-CM codes.
- COVID-19 Questionnaire: At WCV3, actively enrolled parent/caregivers will complete a short (7 item) COVID-19 Questionnaire which asks if they have altered their behavior related to taking their child to the dentist due to the COVID-19 pandemic.

Secondary outcomes and child medical illness (i.e. confounder) will be assessed as follows:

- A newly carious tooth is defined as any tooth receiving an ICDAS lesion code ≥ 3 that received a sound code on the previous dental screening or any tooth surface with an ICDAS filling code ≥ 3 that

previously did not have one. Number of teeth with new decay or filling will be assessed through the clinical dental screenings (T_0, T_3, and T_6) and recorded on the ICDAS Assessment form (Appendix E).

- Questions on frequency of tooth brushing (NHANES III)(CDC), frequency of consumption of sweet food and drinks (NHANES 2009-2010; Youth Risk Behavior Survey 2017)[63,71]
- Early Childhood Oral Health Impact Scale (ECOHIS)[50] will be used to measure oral hygiene, frequency of sweet food and drinks, and oral health-related quality of life (OHRQoL), respectively. Higher scores on the ECOHIS indicate poorer OHRQoL. These questions are included on the A2: Parent Questionnaire (Appendix E) which will be completed at all 3 WCVs (T_0, T_3, and T_6).
- Direct dental costs include costs of preventive and restorative procedures, and dental related ED visits. These costs will be abstracted from Medicaid claims files as part of the verification of child's dental care receipt (described above) and recorded on the Medicaid Claims Abstraction form. Non-dental direct costs (transportation) and dental indirect costs (time away from work, household, or leisure activities) are measured using questions adapted from the Annotated Cost Questionnaire[64] which are incorporated into the B2 Follow-up Questionnaire. The B2 Questionnaire will be administered after WCVs #1 and #2 (T_1 and T_4). Study staff will administer the cost questionnaire at WCV #3 (T_6) to identify any lags in receipt of dental care. Cost data from Medicaid claims files will be abstracted at 3 time points (T_1, T_4, and T_7). See the Annotated Cost Questionnaire and Medicaid Claims Abstraction form (Appendix E) for more details.

Table 8.1.2 Cost elements and sources

Cost Category	Cost Element	Source
Implementation/ training costs	Training providers in communicating oral health facts	Study training records/logs
	IT system modifications	Vendor invoices, clinic personnel time logs
	Staff training	Study staff time/training logs
Intervention costs	Time required to provide education and give prescription	Review of audiotaped encounters
	Cost of dental prescription pads, flip charts, pocket cards	Study/practice expense records
Dental costs	Direct dental costs - Dental utilization costs (including emergency)	Medicaid claims data for participants
	Non-dental direct costs (e.g. transportation to dental visits)	Annotated Cost Questionnaire
	Dental Indirect costs (e.g. time away from work, household, or leisure activities)	Annotated Cost Questionnaire

Note: If training/study staff time logs conflict for a given activity, the arithmetic mean of the two figures will be used in analysis.

- Medical illness data will be abstracted from the child's medical charts (i.e. EMR) and classified into a 10 clinical risk grouping system.[60] EMR abstraction will be performed by study staff after each WCV (T_0, T_3, and T_6). See EMR Abstraction form (Appendix E).

8.1.2 Parent/caregiver

Measures at the parent/caregiver-level are mediators (IPQ-RD and self-efficacy) and moderators (socio-demographic characteristics, health literacy, and social support).

- The Illness Perception Questionnaire-Revised for Dental (IPQ-RD)[72] is comprised of 5 main cognitive constructs (i.e. identity, cause, timeline, consequences, and cure/controllability) and will be used to assess the impact of the provider-level intervention on caregivers' perception of their child's dental caries. Overall and construct-specific scores will be calculated with higher scores indicating caregivers' less accurate/inaccurate perception of the caries disease process and its chronicity. Caregivers will complete the A1: IPQ-RD before the OH encounter with their provider at WCVs #1 - #3 (T_0, T_3, and T_6). The B1: Follow-up IPQ-RD will be completed after WCVs #1 and #2 (T_1 and T_4). See A1: IPQ-RD and B1: Follow-up IPQ-RD in Appendix E.
- Self-efficacy[65] will include 9 items in which parents assess their confidence in their ability to take the child to the dentist despite personal barriers. Questions on self-efficacy are included in the A2: Parent Questionnaire (Appendix E) completed at all 3 WCVs (T_0, T_3, and T_6).
- Moderators include: caregiver health literacy,[73] social support,[66] and socio-demographics,[62] i.e. sex, age, race, education, occupation, and marital status. The A2: Parent Questionnaire (Appendix E) includes questions to collect data on all 3 moderators at all 3 WCVs (T_0, T_3, and T_6).

8.1.3 Provider

Provider-level measures include mediators (OH knowledge and self-efficacy), a confounder (socio-demographic characteristics), and evaluation of implementation/fidelity (observation and audiotape of OH encounters) and adoption & maintenance (i.e. percentage of enrolled patients given a prescription and list of dentists).

- Providers' OH knowledge and self-efficacy to perform OH activities will be assessed through pre- and post-tests (Appendix E) administered before and after the didactic education session prior to WCV #1 (T_{-1}) and booster sessions before WCV #2 (T_2).
- Providers' socio-demographic characteristics (Appendix E) will be collected at the didactic training sessions prior to WCV#1 (T_{-1}).
- As an evaluation of the implementation/fidelity of the intervention, providers' oral health encounters will be observed or audiotaped by study staff and evaluated using the Parent Provider Communication Checklist (Appendix E), the same standardized skills checklist used during the skills training session (T_0, T_3, and T_6).
- Parent/caregivers will evaluate their provider's oral health communication and delivery of prescription and list of dentists on the A3: Parent exit questionnaire (Appendix E) completed at WCVs #1 - #3 (T_0, T_3, and T_6). This is also an evaluation of implementation/fidelity.

- Providers' EMR documentation of prescriptions and lists of dentists given will be audited throughout the main trial (T_0, T_3, and T_6), based on study arm. The percentage of enrolled caregivers who received the prescription and list of dentists will be calculated as an evaluation of providers' adoption and maintenance of the provider-level intervention and recorded on the EMR Audit of Practice form (Appendix E).

- Providers will complete structured interviews with a trained moderator on implementation barriers/enablers to evaluate best strategies for dissemination of the oral health intervention. The interviews will be conducted at the pediatric practices at a time convenient for the provider and will contain interview questions to assess relative advantage of the OH intervention, compatibility of the OH intervention with the needs of the adopters, complexity of the OH intervention, trialability, and observability. Interviews will be conducted using zoom if in-person interviews are not possible due to the current COVID-19 outbreak.

8.1.4 Practice

Practice-level measures include mediators (organization readiness, organizational climate, and practice attitude) and evaluation of adoption and maintenance (percentage of enrolled patients with complete oral health documentation in EMR).

- Mediators of organization readiness and organization climate[69] and practice attitudes toward adopting evidence-based interventions (EBI)[70] will be assessed on the Practice Questionnaire (Appendix E) among leaders and providers from all 18 practices prior to enrollment of caregiver participants (T_{-1}).

- Audits of providers' documentation of all 4 questions in EMR will be performed throughout the main trial (T_0, T_3, and T_6), based on study arm. The percentage of enrolled patients with complete documentation (i.e. all 4 questions) will be recorded on the EMR Audit of Practice form (Appendix E) and serve as an evaluation of the practice's adoption and maintenance of systematic documentation of OH in EMR for Arm A.

9 ASSESSMENT OF SAFETY

9.1 Specification of Safety Parameters

The principal investigator (PI) will report safety events for the study (unanticipated problems [UPs], adverse events [AEs], serious adverse events [SAEs]) to the Institutional Review Board (IRB) in accordance with the IRB's requirements as outlined below.

The PI will also report UPs involving risks to subjects to NIDCR. This will include UPs that meet the definition of a serious adverse event.

9.1.1 Unanticipated Problems

The Office for Human Research Protections (OHRP) considers unanticipated problems involving risks to subjects or others to include, in general, any incident, experience, or outcome that meets **all** of the following criteria:

- unexpected in terms of nature, severity, or frequency given (a) the research procedures that are described in the protocol-related documents, such as the IRB-approved research protocol and informed consent document; and (b) the characteristics of the subject population being studied;
- related or possibly related to participation in the research (“possibly related” means there is a reasonable possibility that the incident, experience, or outcome may have been caused by the procedures involved in the research); and
- suggests that the research places subjects or others at a greater risk of harm (including physical, psychological, economic, or social harm) than was previously known or recognized.

9.1.2 Adverse Events

OHRP defines an adverse event as any untoward or unfavorable medical occurrence in a human subject, including any abnormal sign (for example, abnormal physical exam or laboratory finding), symptom, or disease, temporally associated with the subject’s participation in the research, whether or not considered related to the subject’s participation in the research, (modified from the definition of adverse events in the 1996 International Conference on Harmonization E-6 Guidelines for Good Clinical Practice).

9.1.3 Serious Adverse Events

A serious adverse event (SAE) is one that meets one or more of the following criteria:

- Results in death
- Is life-threatening (places the subject at immediate risk of death from the event as it occurred)
- Results in inpatient hospitalization or prolongation of existing hospitalization
- Results in a persistent or significant disability or incapacity
- Results in a congenital anomaly or birth defect

An important medical event that may not result in death, be life threatening, or require hospitalization may be considered an SAE when, based upon appropriate medical judgment, the event may jeopardize the subject and may require medical or surgical intervention to prevent one of the outcomes listed in this definition.

9.2 Time Period and Frequency for Event Assessment and Follow-Up

Unanticipated problems, AE and SAEs will be recorded in the data collection system throughout the study. Events will be followed for outcome information until resolution or stabilization.

Given the nature of the interventions (EMR changes, education with skills training, and prescription + list of dentists) it is unlikely that intervention-related SAEs or AEs will occur. However, throughout the study, if a caregiver or provider reports an event to any study staff; it will be communicated to the PI

immediately. The PI will record all reportable events occurring any time after informed consent is obtained until the last day of study participation.

9.3 Characteristics of an Adverse Event

9.3.1 Relationship to Study Intervention

To assess relationship of an event to study intervention, the following guidelines are used:

1. Related (Possible, Probable, Definite)
    a. The event is known to occur with the study intervention.
    b. There is a temporal relationship between the intervention and event onset.
    c. The event abates when the intervention is discontinued.
    d. The event reappears upon a re-challenge with the intervention.
2. Not Related (Unlikely, Not Related)
    a. There is no temporal relationship between the intervention and event onset.
    b. An alternate etiology has been established.

9.3.2 Expectedness of SAEs

Given the nature of the interventions (EMR changes, education with skills training, and prescription + list of dentists) it is unlikely that intervention-related SAEs or AEs will occur. The PI is initially responsible for determining SAE/AE expectedness, and NIDCR medical monitor/DSMB will then review and make their determination. An adverse event will be considered unexpected if the nature, severity, or frequency of the event is not consistent with the risk information previously described for the intervention.

9.3.3 Severity of Event

The following scale will be used to grade adverse events:

1. Mild: no intervention required; no impact on activities of daily living (ADL)
2. Moderate: minimal, local, or non-invasive intervention indicated; moderate impact on ADL
3. Severe: significant symptoms requiring invasive intervention; subject seeks medical attention, needs major assistance with ADL

9.4 Reporting Procedures

9.4.1 Unanticipated Problem Reporting to IRB and NIDCR

Incidents or events that meet the OHRP criteria for unanticipated problems require the creation and completion of an unanticipated problem report form. The following information will be included when reporting an adverse event, or any other incident, experience, or outcome as an unanticipated problem to the IRB:

- appropriate identifying information for the research protocol, such as the title, investigator's name, and the IRB project number;
- a detailed description of the adverse event, incident, experience, or outcome;
- an explanation of the basis for determining that the adverse event, incident, experience, or outcome represents an unanticipated problem;
- a description of any changes to the protocol or other corrective actions that have been taken or are proposed in response to the unanticipated problem.

To satisfy the requirement for prompt reporting, unanticipated problems will be reported using the following timeline:

- All unanticipated problems involving risks to subject or others must be reported to the UHCMC IRB within fourteen (14) calendar days of discovery of the problem or event. The only exception to the above timeframe is for the reporting of deaths. All internal, unexpected, study-related deaths must be reported to the IRB within seven calendar days of their discovery. Reporting to NIDCR and the CC must also occur within 7 calendar days if death or a life-threatening event occurs or within 14 calendar days for all other SAEs.

The following are examples of unanticipated problems that need to be reported by the PI to the UHCMC IRB as soon as possible, but in all cases within 14 calendar days:

- Adverse events, which in the opinion of the PI, are **both** unexpected and related or possible related.
    - An adverse event is "unexpected" when its specificity and severity are not accurately reflected in the IRB approved informed consent document.
    - An adverse event is "related to the research procedures" if in the opinion of the PI, it was more likely than not to be caused by the research procedures, or if it more likely than not that the event affects the rights and welfare of current participants.
    - All fatal events determined to be not study-related and expected should be kept in the PI's file and do not need to be reported to the IRB.
    - All fatal events determined to be not study-related and unexpected must be reported to the IRB at the next continuing review or study closure, whichever is first.
- Information that indicates a change to the risks or potential benefits of the research.
- A breach of confidentiality including inappropriate disclosure, lost or stolen confidential information.
- Changes to the protocol taken without prior IRB review to eliminate apparent immediate hazard to a research participant.
- Incarceration of a participant in a protocol not approved to enroll prisoners.
- Event that requires prompt reporting to the sponsor such as disqualification or suspension of investigator.
- Complaint of a participant when the complaint indicates unexpected risks or the complaint cannot be resolved by the research team.

Protocol deviation (including accidental or intentional protocol deviation) that caused harm to participants or others or indicates participants or others are at increased risk of harm. All UPs should be reported to appropriate institutional officials (as required by an institution's written reporting procedures), the supporting agency head (or designee), and OHRP within one month of the IRB's receipt of the report of the problem from the PI.

All UPs will be reported to NIDCR's centralized reporting system via Rho Product Safety, and to the CC:

- Product Safety Fax Line (US): 1-888-746-3293
- Product Safety Fax Line (International): 919-287-3998
- Product Safety Email: rho_productsafety@rhoworld.com
- CC Safety Email: OHDCSAE@ucsf.edu
- CC Safety Fax Line: 1-415-502-8447

General questions about UP reporting can be directed to the Rho Product Safety Help Line (available 8:00AM – 5:00PM Eastern Time):

- US: 1-888-746-7231
- International: 919-595-6486

9.4.2 Serious Adverse Event Reporting to NIDCR

Any AE meeting the specified Serious Adverse Event criteria will be submitted on an SAE form to NIDCR's centralized safety system via Rho Product Safety. This report may be sent by fax or email. Once submitted, Rho Product Safety will send a confirmation email to the investigator within 1 business day. The investigator should contact Rho Product Safety if this confirmation is not received. This process applies to both initial and follow-up SAE reports.

SAE Reporting Contact Information:

- Product Safety Fax Line (US): 1-888-746-3293
- Product Safety Fax Line (International): 919-287-3998
- Product Safety Email: rho_productsafety@rhoworld.com

General questions about SAE reporting can be directed to the Rho Product Safety Help Line (available 8:00AM – 5:00PM Eastern Time):

- US: 1-888-746-7231
- International: 919-595-6486

The study clinician will complete a Serious Adverse Event Form and submit via fax or email within the following timelines:

- All study-related deaths and immediately life-threatening events, will be recorded on the Serious Adverse Event Form and submitted to Product Safety within 7 calendar days of site awareness.

- Study-related serious adverse events other than death and immediately life-threatening events, will be reported by fax within 14 calendar days of site awareness.

All SAEs will be followed until resolution or stabilization.

10 STUDY OVERSIGHT

In addition to the PI’s responsibility for oversight, study oversight will be under the direction of a Data and Safety Monitoring Board (DSMB) composed of members with appropriate clinical, statistical, scientific, and ethical expertise. The NIDCR will appoint the Board. The DSMB will meet at least annually to assess safety and effectiveness data, study progress, and data integrity for the study. If concerns arise, more frequent meetings may be held. The DSMB will operate under the rules of an NIDCR-approved charter that will be approved at the organizational meeting of the DSMB. The DSMB will provide recommendations to the NIDCR.

11 CLINICAL SITE MONITORING

Clinical site monitoring is conducted to ensure that the rights of human subjects are protected, that the study is implemented in accordance with the protocol and/or other operating procedures, and that the quality and integrity of study data and data collection methods are maintained. Monitoring for this study will be performed by NIDCR's Clinical Research Operations and Management Support (CROMS) contractor. The monitor will evaluate study processes and documentation based on NIDCR standards and the International Conference on Harmonisation (ICH), E6: Good Clinical Practice guidelines (GCP).

Details of clinical site monitoring will be documented in a Clinical Monitoring Plan (CMP) developed by the CROMS contractor, in collaboration with the NIDCR Office of Clinical Trials and Operations Management (OCTOM) and the NIDCR Program Official. The CMP will specify the frequency of monitoring, monitoring procedures, the level of clinical site monitoring activities (e.g., the percentage of subject data to be reviewed), and the distribution of monitoring reports. Some monitoring activities may be performed remotely, while others will take place at the study site(s). Staff from the CROMS contractor will conduct monitoring activities and provide reports of the findings and associated action items in accordance with the details described in the CMP. Documentation of monitoring activities and findings will be provided to the site study team, the study PIs, OCTOM, and the NIDCR. The NIDCR reserves the right to conduct independent audits as necessary.

12 STATISTICAL CONSIDERATIONS

12.1 Study Hypotheses

We hypothesize that providers with improved knowledge regarding the chronicity of caries and appropriate skills training (through bundled provider- and practice-level interventions) will deliver consistent reinforcing oral health facts to parents (at annual WCVs) that will result in increased dental utilization for their child compared to those providers delivering usual care based on AAP education.

12.2 Sample Size Considerations

The study design will involve a cluster randomization to two arms:

1. Bundled (Practice + provider) interventions, 2. Enhanced usual care. The assumed response rates (proportion of children who have dental attendance) are Arm A: 0.46 (The dental utilization reported among Medicaid-enrolled children by Vujicic and Nasseh, 2015[74] was 48%, but we used 46% to be conservative), Arm B: 0.30 (based on pilot study where 30% of parent reported routine dental visit for child in the past year).

Eighteen practices (comprising the clusters) will be included in the study. Practices will be randomized to one of the two arms using a restricted randomization scheme with the constraint that 9 practices will be assigned to each arm. This approach will involve the computation of a balance score (for each candidate randomization) based on marginal differences in three key practice-level variables: % Medicaid-enrolled patients [20% to 40% and >40%], ratio of patients to providers, county [Cuyahoga vs. other]). We use the first generated (constrained) randomization that provides good balance according to a pre-selected balance criterion; it may be reasonable to require perfect marginal balance, if (as we expect) there are a reasonably large number of randomizations that yield this.

The primary objective is to compare dental care attendance between the two arms. For simplicity in the power calculation we consider the binary outcome of any dental attendance in the two year period of follow-up. Dental attendance will be based on a single summary score (number of years - out of two - in which the child visits the dentist at least once a year). We thus suppose the use of a two-sided 0.05 alpha level *z* test (with pooled variance) for a difference in proportions. We assume, as a rough approximation, that the study will recruit an equal number of patients in each practice. Making conservative allowances for an intra-cluster (within-practice) correlation (ICC: in the binary dental attendance outcome) of 0.04 and a 25% drop-out rate, a sample size of 512 participants per arm (total n= 1024) will provide an estimated 80% power to detect the above difference in proportions. Alternatively, this sample size (actually 438 per group) will provide 90% power to detect this same effect size if the ICC is only 0.02. If the intervention effect is only 0.12 (0.42 versus 0.30), a sample size of 457 per group (914 total) will still provide 80% power with a dropout rate of 25% if the ICC is only 0.01. For the purposes of this study, a sample size of 512/arm (n=1024) will be recruited.

For sample size estimates, an important secondary outcome will be incidence of new decayed or filled teeth (dft). Mean dft is hypothesized to decrease for Arm A relative to Arm B. Our previous data[75] among 5 to 7 year old low-income children suggests a mean dft for usual care of 4.5 (standard deviation = 7) which we assume to be a reasonable value for the enhanced usual care arm in the present study. We

assume the same standard deviation for the new intervention group, assuming normally distributed responses for the power calculation. In fact, as dft is a count (possibly following a distribution such as Poisson or negative binomial) it is likely that the standard deviation will decrease with the mean, making our approach conservative.[76]

We suppose the use of a two-sided 0.05 alpha level *t* test, to test for a difference in mean dft between the two groups. As done for the primary outcome, we assume an equal number of patients in each practice and allow for 25% dropout. For dft, a robust clinical outcome, we use a smaller ICC of 0.02. Then for the planned recruitment sample of 512 per arm, we will have 74% power to detect a 40% decrease in the mean dft (i.e., a mean dft of 2.7 in the Bundled intervention arm). If the ICC is only 0.1, this power will increase to 84%.

12.3 Planned Interim Analysis

No interim analysis is planned.

12.4 Final Analysis Plan

Primary Statistical Analysis:

Descriptive statistics will include frequencies (categorical variables) and means (continuous variables) of baseline covariates among the three intervention groups. Chi-square tests (for categorical variables) and t- or F-tests (via ANOVA for continuous variables) will be performed in a preliminary analysis to test for group differences.

For the primary outcome, we will use as an overall dental attendance score the number of years (over the two years of follow-up) in which the child visited the dentist. This will be an ordinal outcome with possible scores of 0, 1 or 2. To assess the intervention effect, we will use a generalized estimating equations (GEE) approach, with practices as clusters, based on a proportional odds marginal model. The model covariates will include an intervention indicator variable (equal to 1 for bundled intervention, 0 for enhanced usual care) and a set of baseline variables representing potential confounders. A standard error correction (for example, the method by Morel et al.)[77] will be used to adjust for a small number of clusters and 95% confidence intervals will be computed. This will be an intent-to treat analysis as all randomized participants providing the necessary measurements – regardless of any lack of compliance – will be included in the analysis.

In the event of missing data (for either year) for dental attendance, we will conduct sensitivity analyses by imputing responses under conservative assumptions (favoring the null hypothesis) and re-running the analysis described above on the completed data.

Analysis of Secondary Outcomes: Summary statistics (including means and standard errors) for secondary outcomes will be calculated by intervention group. The same approach as above will be used for binary or ordinal secondary outcomes (oral hygiene, frequency of sweet snacks and beverages). Namely, ordinal outcomes for each variable will be defined that summarize outcomes over time. For continuous outcomes (e.g., OH quality of life, cost), the above method will be modified by using a linear model (identify link) for GEE, modeling the mean response over time as a summary measure. These outcomes will each be tested for normality using the Shapiro-Wilk statistic; outcomes for which normality is violated will be transformed where appropriate or an alternative model used. For count outcomes (e.g., dft accumulated over time), we will use a loglinear model (log link) assuming a negative

binomial or other appropriate (e.g., zero-inflated negative binomial) distribution. For proportion outcomes (e.g., dt/dft), we will use GEE with a logit link, assuming the proportion follows a beta binomial or zero-inflated beta binomial distribution.[77] Similarly, we will fit appropriate GEE models to test for the effect of each implementation strategy on the corresponding outcome (e.g., % prescriptions given as a measure of adoption). As in the dental attendance analysis, the intervention indicator as well as pertinent baseline variables will be included in the model. In addition, interaction terms (baseline variables by intervention) will be included to test for possible effect modification.

For secondary analyses, a GLIMMIX model approach will be considered as an alternative, which may more easily allow for more than one cluster level if needed. Another alternative approach is to model the repeated measurements (again using GEE or GLIMMIX) which will add an additional cluster level – namely, for individuals). Goodness of fit of alternative models will be compared using QIC for GEE (or AIC for GLIMMIX).

In the likely event of missing responses, we will first assess (In the context of repeated measures analyses) whether the data are missing completely at random (MCAR), that is, whether missingness of the given outcome is dependent only on participant baseline characteristics and not further on the observed outcome at an earlier time. This will be done by modeling missing data indicators for the repeated measurements of each outcome using a GEE (or GLIMMIX) approach with a logit link and including appropriate baseline (control) variables and the outcome at the previous time if available. The MCAR null hypothesis will be rejected if the previous outcome has a statistically significant effect on the probability of missing. A nonsignificant effect would support the use of GEE (which assumes MCAR). The missing at random (MAR) assumption (that missing is not related to unobserved measurements), which is made in the GLIMMIX approach or under certain multiple imputation approaches, is not readily tested empirically, but will be evaluated via scientific/contextual considerations.

If the MCAR assumption is found to be violated we will use a multiple imputation (MI) approach in conjunction with the given GLIMMIX or GEE analysis using a suitable imputation model (i.e., using nonmissing outcome measurements as predictors of missing values). MI may also be considered if the MAR assumption is questioned for either GLIMMIX or GEE analyses.

In addition to assessing the overall effects of the interventions, we will investigate the mechanisms (or paths) through which interventions impact dental attendance. As represented in Figure 4.2.1, we hypothesize that significant portions of the intervention effect will occur through each of several paths. For example, intervention may affect provider variables (such as knowledge and self-efficacy) or practice variables (such as EMR documentation, practice readiness, climate, and attitude) which may in turn affect caregiver illness representation which may in turn affect child dental attendance. Alternatively, provider variables may affect child dental attendance via implementation outcomes. Summary measures may be used for some related mediators. For example, we will consider an overall average score for the multiple item scores for caregiver illness representation. We will also include and assess mediators that will help reveal the importance of each of the multiple components of the bundled intervention. For example, we can estimate the proportion of intervention effect mediated by 'compliance' with regard to EMR documentation. A relatively high mediation proportion will suggest efficacy of the corresponding component.

We have previously developed a generalized causal mediation (GCM) analysis methodology and an accompanying R package[78-81] which we will use to estimate and test each path effect and its proportion

of the total intervention effect. The GCM method allows mediators and final outcomes of different types, including continuous, binary, and count (assumed to have normal, Bernoulli, and Poisson or negative binomial distributions, respectively). These options will accommodate the mediators and final outcome (dental attendance, summarized as a binary response) in our model. Each outcome (including mediators) is modeled using a generalized linear model that includes as covariates possible baseline confounders as well as causally preceding variables according to the causal model (as in Figure 4.2.1). The method uses an extended version of the mediation formula and assumes an extended version of the sequential ignorability assumption.[79] Bootstrap resampling is used to compute confidence intervals and a cluster bootstrap option is available to account for unexplained between-cluster (e.g. provider) variability.

Adjustment for Baseline Characteristics: Estimated intervention effects will be adjusted for the following baseline socio-demographic variables: child's age, race, and caregiver SES, education, and marital status.

Model Assumptions: We will assume that the probabilities of dental care receipt follow a logistic regression model with the included covariates and random effects. Per the standard approach, random effects will be assumed to be independent, normally distributed, and outcomes assumed conditionally independent given the random effects. If multiple random effects (e.g., for different cluster levels) are used, they will be assumed to be independent. For secondary outcomes, analogous assumptions, including the assumption of the correct link function, will be made.

Evaluations for the development of the Tool Kit: We will develop a tool kit containing the oral health didactic and skills curriculum, and resources (for providers and parents) that can be distributed to professional organizations (AAP, AAPD, ADA, AANP). This tool kit will be based on the results of the cRCT and will contain OH activities that have demonstrated to be effective, have content that is easy to tailor and apply for stakeholders beyond the study participants, and a table of contents for navigation to the relevant content. Specifically, the results of the cRCT (effectiveness of arm A vs. arm B), process outcomes from the cRCT (provider adoption, implementation metrics such as score cards of EMR audits and parent exit survey, cost-effectiveness analysis) will be combined with the structured interview data that will be collected from the providers to evaluate the dissemination/diffusion strategy to be used for the tool kit. The newly developed OH tool kit would be reviewed by a random sample of 20% of participating primary care providers (≈12 providers) to obtain their views and input on whether the tool kit is easy to understand; use; provides the necessary information for the delivery of evidence-based oral health care in primary care settings; and to solicit any changes to be made.

13 SOURCE DOCUMENTS AND ACCESS TO SOURCE DATA/DOCUMENTS

Study staff will maintain appropriate research records for this study, in compliance with ICH E6, Section 4.9 and regulatory and institutional requirements for the protection of confidentiality of subjects. CWRU will permit authorized representatives of NIDCR, the CC at UCSF and regulatory agencies to examine (and when required by applicable law, to copy) research records for the purposes of quality assurance reviews, audits, and evaluation of the study safety, progress, and data validity.

All records with identifiable information will be kept in secured locked storage units or stored in secure online databases. Only local (i.e. study staff) at CWRU, the Medical Monitor/DSMB, and NIDCR staff appointed to the trial will have access to the records. Access by the DSMB and NIDCR staff is for the purposes of quality assurance reviews, audits, and evaluation of the study safety and progress.

Specific original documents and data records include, but are not limited to:

- **Parent Consent chart** contains all participant-identifying information. The chart includes the signed consent form, Consent Documentation form, the Parent Contact Info form, Unanticipated Problem or Serious Adverse Event forms, Telephone Contact log and Parent Reimbursement form. These documents will contain the participant's name and other confidential information (i.e. names of child/children and other family members). (Electronic and Paper Based)
- **Parent/Child Research chart** includes Case Report Forms (CRFs) and the Progress

  Notes Checklist. Examples are: child's ICDAS caries dental assessment form (WCVs #1, #2, & #3); and parent/caregiver's IPQ-RD and Parent Questionnaire (WCVs #1, #2 & #3) along with follow-up questionnaires (WCVs #1 & #2). (Electronic and Paper Based).

  Medical abstraction for child illnesses will be from the EMR of the participating practices. Medicaid dental claims data will be used to assess dental attendance for the child.
- **Provider Consent chart** contains all participant-identifying information for providers. The chart includes the signed consent form, consent documentation form, and provider sociodemographic questionnaire. (Electronic and Paper Based).
- **Provider Research chart** will include the provider training pre- and post-tests, and data from the provider score card from the study staff observations. (Electronic and Paper Based). Structured interview transcripts will also be stored in the provider research chart.
- **Tracking logs (in REDCap)** (Electronic)
- **Electronic Audio Recording** of patient encounter to evaluate implementation of delivery of oral health facts. Recordings of structured interviews will be kept until the interview has been transcribed.
- **Memoranda** (paper-based)

Some case report forms and all abstractions from EMR and Medicaid dental claims will be completed by study staff (Research Assistants, the Research Associate, or Project Coordinator, depending on the event). Exceptions will be the questionnaires (IPQ-RD and Caregiver Questionnaires), Parent Contact

information and Consent form(s) that will be completed by the parent/caregiver. The study staff will complete all provider audit forms. Completion of essential documents will follow the guidelines outlined in Section 10.4 of the MOP for Case Report forms. Data will be handled in accordance with GCP, U.S. federal regulations, local regulations (if applicable), and instructions from NIH. All essential documents should be filled out completely and signed by the study staff collecting or recording the data. When necessary, essential documents (like consent/assent forms) will be reviewed, signed and dated by the principal investigators or study staff designated by the principal investigators.

14 QUALITY CONTROL AND QUALITY ASSURANCE

14.1 Definitions

Quality Assurance (QA)

The process to ensure the quality of the intervention meets the study design expectations.

Quality Control (QC)

A set of routine technical activities to measure and control the quality of the intervention and accuracy of data acquisition as the intervention is being implemented.

14.2 Study Intervention and Study Questionnaires

14.2.1 Quality Assurance Procedures

Study-Site Specific:

Study staff who will recruit and interact with parents/caregivers at the practices, and document study activities will attend in-person training which will incorporate the topics of human subject protection, Good Clinical Practice (GCP), and adherence to the study protocol. As part of tracking and managing study records, staff members will learn how to document activities that occur with subjects, update study documentation and use the tracking and audit logs. To assess proficiency, the study staff will be certified in the topics presented to ensure their comprehension of the expectations.

In addition, all study staff will be trained to make calls during the dedicated staff training. A script for each type of call will be created and adherence to the script for study staff telephone contact with caregivers will be assessed throughout the study to ensure that they have not provided additional information to the children's' caregivers that might influence the outcome. (To be detailed further in MOP section 5).

Additionally, the dental hygienists will undergo calibration exercises prior to the main study to become proficient in exam related study procedures and to maintain intra- and inter-examiner agreement. The gold standard examiner will calibrate/train the examiners in the ICDAS protocol in a 4-day training session. Detailed caries assessment and examiner training, calibration and reproducibility protocols will be outlined in the section 5 of the MOP. Hygienists will not utilize dental radiographs. At the follow-up and final WCV screenings, the hygienists will not have access to the results of the baseline examination to avoid detection bias.

On the practice level, enhancements to the EMR system to include OH will be implemented prior to enrolling any parent/child dyad into the study. Providers will be trained and given a manual for EMR documentation for future reference. Study staff will receive formal training to evaluate providers' oral health encounters (through observation or audiotaping), perform audits of EMR documentation, and provide score card feedback to providers. Prior to enrollment of parent/caregivers, study staff will be trained by the communication specialist in a 60-minute session. Training will focus on evaluation of providers' skills during oral health encounters with caregivers.

In education sessions for the provider, instructors will follow scripts to ensure that the curricula are delivered in the same way for each group of clinicians to ensure fidelity. The training will be conducted

by Drs. Nelson, Ferretti, and Lord. Booster training will be conducted yearly or as needed if there are deviations in protocol.

CC Specific:

Before formal data entry begins, appropriate study staff will be trained on a custom-configured, study-specific Electronic Data Capture (EDC) test system. This procedure is for data entry training and user acceptance testing, concurrently. After completing didactic training, staff trainees will enter pre-specified test data into the test EDC system, including invalid data, to provide additional training and familiarity with the data entry process. Their entered data will be compared against the pre-specified test data. After completion of training, staff members will be granted permission to use the EDC production environment for collection of project data.

14.2.2 Quality Control Procedures

Study-Site Specific:

Data quality control is primarily conducted at the study team level through internal processes of data review/data monitoring as the data is collected and through periodic custom reports generated by the CC (See Table 16.4.1). Dental screening data will be reviewed for accuracy and completeness after each study visit by the Dental Hygienist. The Research Assistants will review the questionnaires after the participants have completed them. Each paper form and questionnaire will be entered and verified by two separate individuals. Forms completed electronically via tablets will be checked by study staff prior to the parent/caregiver leaving the WCV. Any field that is unclear will be clarified with the parent/caregiver who completed the document.

In addition, the first five (5) study staff telephone contacts (reminder calls to return the B1 questionnaires) will be audio recorded in digital media and reviewed. Then, the first five (5) B2 follow-up calls will be recorded and reviewed for each staff member. At the beginning of the study, the Project Coordinator will review the first five telephone contacts to ensure fidelity of the calls and accuracy of data recording. Study staff will be provided with feedback on their performance and conduct including but not limited to these specific areas; adherence to telephone script, adherence to protocol/MOP, adherence to good clinical practice. Study staff with telephone contact with less than acceptable quality will receive a private training session focused on problematic areas. This QC activity will take place until the first five sessions have been completed. This will ensure that any weaknesses in study protocol will be addressed early.

All study forms and questionnaires collected and entered into the database will be checked for inconsistencies and range and assessed for missing data. Any inconsistencies, outliers, or missing data observed will be compared to the paper document and appropriate corrective actions carried out. REDCap’s native data resolution workflow will be used to document and fix any data anomalies. The Data Manager will also respond to data queries generated by the PI, Study Coordinator, or other study staff.

CC Specific:

The CC will assist with the design of custom reports. Utilization of an EDC system, such as REDCap, means that automated data checks can be implemented within the data entry system. This helps to prevent most errors immediately. The automated checks are also supported by an extensive system of

custom reports and manual validation procedures that help to assist in the resolution of any additional errors.

The CC will create a standalone Data Validation Plan (DVP). The DVP will describe in more specific detail the edit-checks that will be performed within the EDC system and the SOPs for data entry and quality management. The CC Clinical Research Specialist and the Data Manager assigned to the specific project, together with the study team, will be responsible for the creation, testing, and finalization of the DVP for the project.

Following collection of all data from a project, additional data processing will be required by the CC, e.g., longitudinal coding of dental examination data, creation of psychosocial scale scores. In conjunction with that data processing, The CC will run regular validation reports to detect data anomalies and will work with the local project staff to resolve any data anomalies that arise during data entry. Following PI concurrence, the database will be locked.

15 ETHICS/PROTECTION OF HUMAN SUBJECTS

15.1 Ethical Standard

The investigator will ensure that this study is conducted in full conformity with the principles set forth in The Belmont Report: Ethical Principles and Guidelines for the Protection of Human Subjects of Research, as drafted by the US National Commission for the Protection of Human

Subjects of Biomedical and Behavioral Research (April 18, 1979) and codified in 45 CFR Part 46 and/or the ICH E6.

15.2 Institutional Review Board

The protocol, informed consent form(s), recruitment materials, and all subject materials will be submitted to the University Hospitals Cleveland Medical Center (FWA#: 00004428) IRB for review and approval. Approval of both the protocol and the consent form must be obtained before any subject is enrolled. Any amendment to the protocol will require review and approval by the IRB before the changes are implemented in the study.

15.3 Informed Consent Process

Informed consent is a process that is initiated prior to the individual agreeing to participate in the study and continues throughout study participation. Extensive discussion of risks and possible benefits of study participation will be provided to subjects and their families, if applicable. A consent form describing in detail the study procedures and risks will be given to the subject. Consent forms will be IRB-approved, and the subject is required to read and review the document or have the document read to him or her. The investigator or designee will explain the research study to the subject and answer any questions that may arise. The subject will sign the informed consent document prior to any study-related assessments or procedures. Subjects will be given the opportunity to discuss the study with their surrogates or think about it prior to agreeing to participate. They may withdraw consent at any time throughout the course of the study. A copy of the signed informed consent document will be given to subjects for their records. The rights and welfare of the subjects will be protected by emphasizing to them that the quality of their clinical care will not be adversely affected if they decline to participate in this study. The consent process will be documented in the research record.

Active participants completing WCV3 that will be asked to complete the COVID-19 Dental Visit Questionnaire will be re-consented with the consent form updated 12/7/20.

All active providers that will be asked to complete a structured interview will be re-consented prior to the structured interview session.

15.4 Subject Confidentiality

Subject confidentiality is strictly held in trust by the investigators, study staff, representatives for University Hospitals Cleveland Medical Center Institutional Review Board, and the representatives of the study sponsor (NIDCR).

The study protocol, documentation, data, and all other information generated will be held in strict confidence. No information concerning the study or the data will be released to any unauthorized third party without prior written approval of the sponsor.

The study monitor or other authorized representatives of the sponsor may inspect all study documents and records required to be maintained by the investigator, including but not limited to, medical records (office, clinic, or hospital) for the study subjects. The clinical study site will permit access to such records.

All information about the provider and parent/caregiver will be kept strictly confidential and be used only for study purposes. When study results are published or presented, all information will be presented in group form, without identifiable information.

16 DATA HANDLING AND RECORD KEEPING

The investigators are responsible for ensuring the accuracy, completeness, legibility, and timeliness of the data reported. All source documents should be completed in a neat, legible manner to ensure accurate interpretation of data. The investigators will maintain adequate case histories of study subjects, including accurate case report forms (CRFs), and source documentation. (Will be detailed in the MOP)

The study staff will collaborate and interact with the CC to perform data management and quality control activities.

16.1 Data Management Responsibilities

Data collection and accurate documentation are the responsibility of the Research Assistants, Data Manager and Study Coordinator under the supervision of the PI. All source documents must be reviewed by the study team and data entry staff, who will ensure that they are accurate and complete. Unanticipated problems and adverse events must be reviewed by the PI.

16.2 Data Capture Methods

Data for this study will be captured using dental screenings, forms, questionnaires, and audio recordings. Study data will be collected and stored using the REDCap platform hosted by University of California-San Francisco, the home institution of the CC. *The UCSF Coordinating Center will end its coordinating activities at the conclusion of their funding period. However, our study activities and funding will go on beyond the time when CC ends its activities, therefore the CC has indicated that it will transfer all data from UCSF REDCap to UHHS REDCap so that the study team can complete its data collection, entry, checking, and analysis activities. The data will be transferred in a secure manner to UHHS REDCap and all data for the study will then reside in UHHS REDCap.* REDCap is a secure, web-based application designed to support remote data capture for research studies, providing: 1) an intuitive interface for validated data entry, 2) audit trails for tracking data manipulation and export procedures, 3) automated export procedures for seamless data downloads to common statistical packages, and 4) procedures for importing data from external sources.

Study forms will be completed by participants on paper, and subsequently entered into REDCap by study staff, or on a tablet directly into REDCap. Paper forms will be securely stored in a locked file cabinet. Recorded audio will be deleted from the digital recording device immediately after being stored on a secure CWRU School of Dental Medicine network drive.

16.3 Types of Data

Data for this study will include: (1) dental screening data, (2) study questionnaires, (3) abstracted medical data, (4) abstracted Medicaid dental claims data, (5) cost data (6) data from observation/audiotaping of providers, (7) EMR audit data, and (8) structured interview transcripts. Additionally, audio recordings will be used for fidelity monitoring and to capture responses from the provider structured interviews. Form revisions should be minimal; however, should they occur, changes will be submitted to the CC for updating and dissemination to study staff.

16.4 Schedule and Content of Reports

Quality control is primarily conducted at the study team level through internal processes of data review/data monitoring using periodic custom reports generated by the CC. The CC will assist with the design of project-specific custom reports. See Table 16.4.1 for the project-specific custom reports for the main trial.

Table 16.4.1 Project-specific custom reports created by CC

**Responsible for Addressing**	**Name of Report**	**Frequency**
CC	Weekly Enrollment	Weekly during recruitment
CC	Cumulative Enrollment	Weekly during recruitment
CWRU	Informed Consent & Documentation Errors	Weekly during recruitment
CWRU	Fidelity Monitoring -Provider Level	As Required by CWRU
CWRU	Fidelity Monitoring - Practice Level	As Required by CWRU
CWRU	B1 Calls	As Required by CWRU
CWRU	B1 Incentive Mailing	As Required by CWRU
CWRU	B2 Calls	As Required by CWRU
CWRU	B2 Incentive Mailing	As Required by CWRU
CWRU	Child Illness EMR Abstraction	As Required by CWRU
CWRU	Audit of OH Documentation	As Required by CWRU
CWRU	WCV to be scheduled	As Required by CWRU

Study-Site Specific:

The CC will run regular validation reports to detect data anomalies and will work with the local project staff to resolve any data anomalies that arise during data entry. REDCap's native data resolution workflow will be used to document and fix any data anomalies. The Data Manager will also respond to data queries generated by the PI, Study Coordinator, or other study staff.

At the completion of the study, the study Biostatistician will conduct analyses of the data and assist in preparation of study publications and presentations. The Data Manager will provide technical and data support for the Biostatistician throughout the study.

Coordinating Center (CC):

The CC will generate regular reports showing enrollment and potential data anomalies, which will be sent to PIs, Project Coordinators, and other relevant study staff. The CC will generate a monthly enrollment report (or as requested by the DSMB or NIDCR Program Official) for sharing progress of the study (see table 16.4 above).

16.5 Study Records Retention

Study documents should be retained for 3 years after the final financial report for the grant. These documents may be retained for a longer period, however, if required by local regulations or UHCMC IRB.

16.6 Protocol Deviations

A protocol deviation is any noncompliance with the clinical study protocol or Good Clinical Practice requirements. The noncompliance may be on the part of the subject, the investigator, or study staff (e.g., Project Coordinators, Research Assistants). As a result of deviations, corrective actions are to be developed by the PI and/or Project Coordinator, and implemented promptly.

These practices are consistent with investigator and sponsor obligations in ICH E6:

- Compliance with Protocol, Sections 4.5.1, 4.5.2, 4.5.3, and 4.5.4.
- Quality Assurance and Quality Control, Section 5.1.1
- Noncompliance, Sections 5.20.1 and 5.20.2.

All deviations from the protocol must be addressed in study subject source documents and promptly reported to NIDCR and the UHCMC IRB, according to their requirements as outlined below

According to UHCMC IRB, Major Deviations are reported to the IRB within 14 calendar days of discovery. Minor Deviations are kept in the investigator's file to be reported at the time of continuing review.

Examples of Major Deviations:

- Failure to obtain informed consent, i.e., there is not documentation of informed consent or informed consent was obtained after initiation of study procedures;
- Informed consent obtained by someone not approved to obtain consent for the protocol;
- Use of invalid consent form, i.e. consent form without IRB approval; or outdated/expired consent form;
- Enrollment of a participant who was ineligible for the study;
- Performing a research procedure not in the approved protocol;
- Failure to report serious adverse event to IRB; sponsor; and/or regulatory agencies;
- Failure to follow the approved study protocol that affect participant safety or data integrity (e.g., study visit missed or conducted outside of required timeframe, or failure to perform a laboratory test);
- Failure to follow safety monitoring plan;
- Continuing research activities after IRB approval has expired;
- Use of recruitment procedures that have not been approved by the IRB;
- Enrolling significantly more subjects than proposed in the IRB protocol;

Examples of Minor Deviations:

- Missing original signed and dated consent form (only a photocopy available);
- Missing pages of executed consent form;

- Failure to follow the approved study protocol that does not affect participant safety (e.g., study procedure conducted out of sequence, failure to perform a required test, missing laboratory results, study visit conducted outside of required timeframe.).

17 PUBLICATION/DATA SHARING POLICY

This study will comply with the *NIH Public Access Policy*, which ensures that the public has access to the published results of NIH funded research. It requires scientists to submit final peer-reviewed journal manuscripts that arise from NIH funds to the digital archive PubMed Central upon acceptance for publication.

Data from this study will be shared in accordance with the NIH Data Sharing Policy.

https://grants.nih.gov/grants/policy/data_sharing/data_sharing_guidance.htm

This study will be registered and results updated periodically through ClinicalTrials.gov in accordance with the NIH Policy on the Dissemination of NIH-Funded Clinical Trial Information to promote broad and responsible dissemination of information from NIH-funded clinical trials.

https://www.federalregister.gov/documents/2016/09/21/2016-22379/nih-policy-on-thedissemination-of-nih-funded-clinical-trial-information

18 LITERATURE REFERENCES

1. U.S. Department of Health and Human Services. Oral Health in America: A Report of the Surgeon General, Rockville, MD: U.S. Department of Health and Human Services, National Institute of Dental and Craniofacial Research, National Institutes of Health, 2000. Retrieved from http://www.surgeongeneral.gov/library/oralhealth/

2. Dye, BA, Arevalo, O, Vargas, CM. Trends in paediatric dental caries by poverty status in the United States, 1988-1994 and 1999-2004. *Int J Paediatr Dent. 2010; 20*(2), 132–143. https://doi.org/10.1111/j.1365-263X.2009.01029.x

3. Dye, BA. The Global Burden of Oral Disease: Research and Public Health Significance. *J Dent Res*. 2017; 96(4):361-363. doi:10.1177/0022034517693567.

4. Nelson, S, Mandelaris, J, Ferretti, G, Heima, M, Spiekerman, C, Milgrom, P. School screening and parental reminders in increasing dental care for children in need: a retrospective cohort study. *J Public Health Dent.* 2012; *72*(1), 45–52. https://doi.org/10.1111/j.1752-7325.2011.00282.x

5. Dye, BA, Thornton-Evans, G, Li, X, Iafolla, TJ. Dental Caries and Tooth Loss in Adults in the United States, 2011–2012. *NCHS Data Brief.* 2015; (197), 1–8. https://doi.org/10.1080/713604647

6. Hakim, RB, Babish, JD, Davis, AC. State of dental care among Medicaid-enrolled children in the United States. *Pediatrics.* 2012; *130*(1), 5–14. https://doi.org/10.1542/peds.20112800

7. United States General Accounting Office. Medicaid: Extent of dental disease in children has not decreased, and millions are estimated to have untreated tooth decay. 2008. *(Report Number GAO-08-1121). Wahsington, DC*.

8. Yarbrough, C, Nasseh, K, Vujicic, M. Key Differences in Dental Care Seeking Behavior between Medicaid and Non- Medicaid Adults and Children. 2014. Retrieved from http://www.ada.org/~/media/ADA/Science and Research/HPI/Files/HPIBrief_0814_4.ashx

9. Divaris, K, Lee, JY, Baker, AD, Gizlice, Z, Rozier, RG, DeWalt, DA, Van, WF Jr. Influence of Caregivers and Children's Entry Into the Dental Care System. *Pediatrics.* 2014; *133*(5), e1268-76. https://doi.org/10.1542/peds.2013-2932

10. American Academy of Pediatric Dentistry. Guideline On Infant Oral Health Care. *Pediatric Dentistry.* 2014; *37*(6), 146–150.

11. U.S. Public Health Service Dept. of Health and Human Services. The Department of Health and Human Services 2015 Annual Report on the Quality of Care for Children in Medicaid and CHIP Health and Human Services Secretary. 2015. Retrieved from https://www.medicaid.gov/medicaid/quality-of-care/downloads/2015-child-sec-rept.pdf

12. Chou, R, Cantor, A, Zakher, B, Mitchell, JP, Pappas, M. *Prevention of Dental Caries in Children Younger Than 5 Years Old*. *Prevention of Dental Caries in Children Younger Than 5 Years Old: Systematic Review to Update the U.S. Preventive Services Task Force Recommendation*. 2014. Agency for Healthcare Research and Quality (US). Retrieved from http://www.ncbi.nlm.nih.gov/pubmed/24872964

13. Kranz, AM, Preisser, JS, Rozier, RG. Effects of Physician-Based Preventive Oral Health Services on Dental Caries. *PEDIATRICS. 2015; 136*(1), 107–114. https://doi.org/10.1542/peds.2014-2775

14. Kranz, AM, Lee, J, Divaris, K, Baker, AD, Vann, W. North Carolina physician based preventive oral health services improve access and use among young Medicaid enrollees. *Health Affairs (Project Hope).* 2014; *33*(12), 2144–52. https://doi.org/10.1377/hlthaff.2014.0927

15. Kranz, AM, Rozier, RG, Preisser, JS, Stearns, SC, Weinberger, M, Lee, JY. Comparing medical and dental providers of oral health services on early dental caries experience. *American Journal of Public Health.* 2014; *104*(7), e92-9. https://doi.org/10.2105/AJPH.2014.301972

16. Blackburn, J, Morrisey, MA, Sen, B. Outcomes Associated With Early Preventive Dental Care Among Medicaid-Enrolled Children in Alabama. *JAMA Pediatrics.* 2017; *171*(4), 335. https://doi.org/10.1001/jamapediatrics.2016.4514

17. Milgrom, PM, Cunha-Cruz, J. Are Tooth Decay Prevention Visits in Primary Care Before Age 2 Years Effective? *JAMA Pediatrics.* 2017; *171*(4), 321. https://doi.org/10.1001/jamapediatrics.2016.4982

18. Beauchamp, J, Caufield, PW, Crall, JJ, Donly, K, Feigal, R, Gooch, B, Ismail, A, Kohn, W, Siegel, M, Simonsen, R. American Dental Association Council on Scientific, A. (2008). Evidence-based clinical recommendations for the use of pit-and-fissure sealants: a report of the American Dental Association Council on Scientific Affairs. *J Am Dent Assoc*. 2008; *139*(3), 257–268. Retrieved from http://www.ncbi.nlm.nih.gov/pubmed/18310730

19. Casamassimo, PS, Lee, JY, Marazita, ML, Milgrom, P, Chi, DL, Divaris, K. Improving children's oral health: an interdisciplinary research framework. *J Dent Res.* 2014; *93*(10), 938–942. https://doi.org/10.1177/0022034514547273

20. Griffin, SO, Oong, E, Kohn, W, Vidakovic, B, Gooch, BF, Bader, J, Clarkson, J, Fontana, MR, Meyer, DM, Rozier, RG, Weintraub, JA, Zero, DT. The Effectiveness of Sealants in Managing Caries Lesions. *Journal of Dental Research.* 2008*; 87*(2), 169–174. https://doi.org/10.1177/154405910808700211

21. Kay, EJ, Locker, D. Is dental health education effective? A systematic review of current evidence. *Community Dent Oral Epidemiol.* 1996; *24*(4), 231–235. Retrieved from http://www.ncbi.nlm.nih.gov/pubmed/8871028

22. Lee, JY, Divaris, K. The ethical imperative of addressing oral health disparities: a unifying framework. *J Dent Res. 2014; 93*(3), 224–230. https://doi.org/10.1177/0022034513511821

23. Milgrom, P, Zero, DT, Tanzer, JM. An examination of the advances in science and technology of prevention of tooth decay in young children since the Surgeon General's Report on Oral Health. *Acad Pediat. 2009; 9*(6), 404–409. https://doi.org/10.1016/j.acap.2009.09.001

24. Sgan-Cohen, HD, Evans, RW, Whelton, H, Villena, RS, MacDougall, M, Williams, DM, IADR Steering and Task Groups. IADR Global Oral Health Inequalities Research Agenda (IADRGOHIRA(R)): a call to action. *J Dent Res. 2013; 92*(3), 209–211. https://doi.org/10.1177/0022034512475214

25. Hooley, M, Skouteris, H, Boganin, C, Satur, J, Kilpatrick, N. Parental influence and the development of dental caries in children aged 0-6 years: a systematic review of the literature. *J Dent.* 2012; *40*(11), 873–885. https://doi.org/S0300-5712(12)00200-X [pii]10.1016/j.jdent.2012.07.013

26. Leventhal, H, Benyamini, Y, Brownlee, S, Diefenbach, M, Leventhal, EA, Patrick-Miller, L, Robitaille, C. Illness representations: theoretical foundations. (W. J. A. Petrie KJ, Ed.), *Perceptions of Health and Illness*. 1997. Amsterdam: Harwood Academic.

27. Leventhal, H, Brissette, I. The common-sense model of self-regulation of health and illness. In L. H. Cameron LD (Ed.), *The self-regulation of health and illness behavior* (pp. 42–65). 2003. London: Routledge.

28. Moss-Morris, R, Weinman, J, Petrie, KJ, Horne, R, Cameron, LD, Buick, D. The Revised Illness Perception Questionnaire (IPQ-R). *Psychology and Health. 2002; 17*(1), 1–16. https://doi.org/Doi 10.1080/08870440290001494

29. Edgar, KA, Skinner, TC. Illness Representations and Coping as Predictors of Emotional Well-being in Adolescents with Type 1 Diabetes. *Journal of Pediatric Psychology. 2003; 28*(7), 485–493. https://doi.org/10.1093/jpepsy/jsg039

30. Hagger, MS, Orbell, S. A Meta-Analytic Review of the Common-Sense Model of Illness Representations. *Psychology & Health. 2003;18*(2), 141–184. https://doi.org/10.1080/088704403100081321

31. Abubakari, AR, Jones, MC, Lauder, W, Kirk, A, Devendra, D, Anderson, J. Psychometric properties of the Revised Illness Perception Questionnaire: factor structure and reliability among African-origin populations with type 2 diabetes. *Int J Nurs Stud. 2012; 49*(6), 672–681. https://doi.org/10.1016/j.ijnurstu.2011.11.008S0020-7489(11)00450-0 [pii]

32. Brink, E, Alsen, P, Cliffordson, C. Validation of the Revised Illness Perception Questionnaire (IPQ-R) in a sample of persons recovering from myocardial infarction--the Swedish version. *Scand J Psychol. 2011; 52*(6), 573–579. https://doi.org/10.1111/j.14679450.2011.00901.x

33. Cabassa, LJ, Lagomasino, IT, Dwight-Johnson, M, Hansen, MC, Xie, B. Measuring Latinos' perceptions of depression: a confirmatory factor analysis of the Illness Perception Questionnaire. *Cultur Divers Ethnic Minor Psychol. 2008; 14*(4), 377–384. https://doi.org/10.1037/a0012820

34. Chen, S L, Tsai, JC, Lee, WL. Psychometric validation of the Chinese version of the Illness Perception Questionnaire-Revised for patients with hypertension. *J Adv Nurs. 2008; 64*(5), 524–534. https://doi.org/10.1111/j.1365-2648.2008.04808.x

35. Hurt, CS, Burns, A, Brown, RG, Barrowclough, C. Perceptions of subjective memory complaint in older adults: the Illness Perception Questionnaire-Memory (IPQ-M). *Int Psychogeriatr. 2010; 22*(5), 750–760. https://doi.org/10.1017/S1041610209991542

36. Lobban, F, Barrowclough, C, Jones, S. Assessing cognitive representations of mental health problems. I. The illness perception questionnaire for schizophrenia. *Br J Clin Psychol. 2005; 44*(Pt 2), 147–162. https://doi.org/10.1348/014466504X19497

37. Covic, A, Seica, A, Gusbeth-Tatomir, P, Gavrilovici, O, Goldsmith, DJ. Illness representations and quality of life scores in haemodialysis patients. *Nephrol Dial Transplant. 2004; 19*(8), 2078–2083. https://doi.org/10.1093/ndt/gfh254

38. Fowler, C, Baas, LS. Illness representations in patients with chronic kidney disease on maintenance hemodialysis. *Nephrol Nurs J.* 2006; *33*(2), 173-174-186. Retrieved from http://www.ncbi.nlm.nih.gov/pubmed/16613412

39. Giannousi, Z, Manaras, I, Georgoulias, V, Samonis, G. Illness perceptions in Greek patients with cancer: a validation of the Revised-Illness Perception Questionnaire. *Psychooncology. 2010; 19*(1), 85–92. https://doi.org/10.1002/pon.1538

40. Hou, R, Cleak, V, Peveler, R. Do treatment and illness beliefs influence adherence to medication in patients with bipolar affective disorder? A preliminary cross-sectional study. *Eur Psychiatry. 2010; 25*(4), 216–219. https://doi.org/10.1016/j.eurpsy.2009.09.003

41. Davies, MJ, Heller, S, Skinner, TC, Campbell, MJ, Carey, ME, Cradock, S, et al. Effectiveness of the diabetes education and self management for ongoing and newly diagnosed (DESMOND) programme for people with newly diagnosed type 2 diabetes: cluster randomised controlled trial. *BMJ. 2008; 336*(7642), 491–495. https://doi.org/10.1136/bmj.39474.922025.BE

42. Galli, U, Ettlin, DA, Palla, S, Ehlert, U, Gaab, J. Do illness perceptions predict pain-related disability and mood in chronic orofacial pain patients? A 6-month follow-up study. *Eur J Pain.* 2010; *14*(5), 550–558. https://doi.org/10.1016/j.ejpain.2009.08.011

43. Mosleh, SM, Bond, CM, Lee, AJ, Kiger, A, Campbell, NC. Effectiveness of theory-based invitations to improve attendance at cardiac rehabilitation: a randomized controlled trial. *Eur J Cardiovasc Nurs.* 2014; *13*(3), 201–210. https://doi.org/10.1177/1474515113491348

44. Nelson, S, Slusar, MB, Albert, JM, Riedy, CA. Do baby teeth really matter? Changing parental perception and increasing dental care utilization for young children. *Contemporary Clinical Trials. 2017; 59*, 13–21. https://doi.org/10.1016/j.cct.2017.05.002

45. Bartholomew, LK, Mullen, PD. Five roles for using theory and evidence in the design and testing of behavior change interventions. *J Public Health Dent. 2011; 71 Suppl 1*, S20-33. Retrieved from http://www.ncbi.nlm.nih.gov/pubmed/21656946

46. Bandura, A. Social cognitive theory of self-regulation. *Organizational Behavior and Human Decision Processes. 1991; 50*(2), 248–287. https://doi.org/http://dx.doi.org/10.1016/07495978(91)90022-L

47. Greenhalgh T, Robert, G, Bate P, Macfarlane F, Kyriakidou O. *Diffusion of innovations in health service organisations, A systematic literature review*. 2005. Malden, MA: Blackwell Publishing Ltd.

48. Rogers, E. *Diffusion of Innovations*. *Free Press New York. Schaeffer, P*. 2003. Free Press. https://doi.org/citeulike-article-id:126680

49. Beil, HA, Rozier, RG. Primary health care providers' advice for a dental checkup and dental use in children. *Pediatrics. 201; 126*(2), e435-41. https://doi.org/10.1542/peds.20092311

50. Pahel, BT, Rozier, RG, Slade, GD. Parental perceptions of children's oral health: the Early Childhood Oral Health Impact Scale (ECOHIS). *Health and Quality of Life Outcomes. 2007; 5*, 6. https://doi.org/10.1186/1477-7525-5-6

51. McLeroy, KR, Bibeau, D, Steckler, A, Glanz, K. An ecological perspective on health promotion programs. *Health Educ Q. 1988; 15*(4), 351–377. Retrieved from http://www.ncbi.nlm.nih.gov/pubmed/3068205

52. Sallis, JF, Owen, N. Ecological models of health behavior. In Glanz and Lewis (Ed.), *Health Behavior and Health Education: Theory, Research, and Practice, third edition* (pp. 462–484). 2002. San Francisco: Jossey-Bass.

53. Glasgow, RE, McKay, HG, Piette, JD, Reynolds, KD. The RE-AIM framework for evaluating interventions: what can it tell us about approaches to chronic illness management? *Patient Educ Couns. 2001; 44*(2), 119–127. Retrieved from http://www.ncbi.nlm.nih.gov/pubmed/11479052

54. Glasgow, RE, Vogt, TM, & Boles, SM. Evaluating the public health impact of health promotion interventions: the RE-AIM framework. *Am J Public Health. 1999; 89*(9), 1322–1327. Retrieved from http://www.ncbi.nlm.nih.gov/pubmed/10474547

55. Cully, JA, Breland, JY, Robertson, S, Utech, AE, Hundt, N, Kunik, ME, Peterson, NJ, Masozera, N, Rao, RNaik, AD. Behavioral health coaching for rural veterans with diabetes and depression: a patient randomized effectiveness implementation trial. *BMC Health Serv Res. 2014; 14*, 191. https://doi.org/10.1186/1472-6963-14-191

56. Ismail, AI, Nainar, SM, Sohn, W. Children's first dental visit: attitudes and practices of US pediatricans and family physicians. *Pediatr Dent. 2003; 25*(5), 425–430. Retrieved from http://www.ncbi.nlm.nih.gov/pubmed/14649605

57. Vann Jr, WF, Divaris, K, Gizlice, Z, Baker, AD, Lee, JY. Caregivers' health literacy and their young children's oral-health-related expenditures. *J Dent Res. 2013; 92*(7 Suppl), 55S–62S. https://doi.org/10.1177/0022034513484335

58. Duijster, D, van Loveren, C, Dusseldorp, E, Verrips, GH. Modelling community, family, and individual determinants of childhood dental caries. *Eur J Oral Sci. 2014; 122*(2), 125– 133. https://doi.org/10.1111/eos.12118

59. Chi, DL, Momany, ET, Neff, J, Jones, MP, Warren, JJ, Slayton, RL, Weber-Gasparoni, K, Damiano, PC. Impact of chronic condition status and severity on the time to first dental visit for newly Medicaid-enrolled children in Iowa. *Health Serv Res.* 2011; *46*(2), 572–595. https://doi.org/10.1111/j.1475-6773.2010.01172.x

60. Chi, DL, Raklios, NA. The relationship between body system-based chronic conditions and dental utilization for Medicaid-enrolled children: a retrospective cohort study. *BMC Oral Health. 2012; 12*, 28. https://doi.org/10.1186/1472-6831-12-28

61. Pitts, N. "ICDAS"--an international system for caries detection and assessment being developed to facilitate caries epidemiology, research and appropriate clinical management. *Community Dent Health. 2004; 21*(3), 193–198. Retrieved from http://www.ncbi.nlm.nih.gov/pubmed/15470828

62. Centers for Disease Control and Prevention. (n.d.). NHANES - NHANES III. Retrieved June 7, 2017, from https://www.cdc.gov/nchs/nhanes/nhanes3.htm

63. Centers for Disease Control and Prevention. 2017 National Youth Risk Behavior Survey. 2017. Retrieved June 22, 2017, from https://www.cdc.gov/healthyyouth/data/yrbs/pdf/2017/2017_yrbs_national_hs_questionnair e.pdf

64. Thompson, S, Wordsworth, S. An annotated cost questionnaire for completion by patients. 2001. Retrieved June 22, 2017, from http://www.dirum.org/assets/downloads/634432307461163116-An annotated cost questionnaire for completion by patients.pdf

65. Detroit Dental Health Project. Detroit Dental Health Project QUESTIONS FOR EVALUATION OF BEHAVIORAL INTERVENTION (Oral Health Behaviors, Self Efficacy). 2004. Retrieved June 22, 2017, from http://www.dent.umich.edu/sites/default/files/departments/crse/W2_Evaluation_Questionnai re.pdf

66. Zimet, GD, Dahlem, NW, Zimet, SG, Farley, GK. The Multidimensional Scale of Perceived Social Support. *Journal of Personality Assessment. 1988; 52*(1), 30–41. https://doi.org/10.1207/s15327752jpa5201_2

67. Lewis, CW, Boulter, S, Keels, MA, Krol, DM, Mouradian, WE, O'Connor, KG, Quinonez, RB. Oral health and pediatricians: results of a national survey. *Acad Pediatr. 2009; 9*(6), 457–461. https://doi.org/10.1016/j.acap.2009.09.016

68. Quinonez, RB, Kranz, AM, Long, M, Rozier, RG. Care coordination among pediatricians and dentists: a cross-sectional study of opinions of North Carolina dentists. *BMC Oral Health.* 2014; *14*, 33. https://doi.org/10.1186/1472-6831-14-33

69. Stamatakis, KA, McQueen, A, Filler, C, Boland, E, Dreisinger, M, Brownson, RC, Luke, DA. Measurement properties of a novel survey to assess stages of organizational readiness for evidence-based interventions in community chronic disease prevention settings. *Implement Sci. 2012; 7*, 65. https://doi.org/10.1186/1748-5908-7-65

70. Aarons, GA. Mental health provider attitudes toward adoption of evidence-based practice: the Evidence-Based Practice Attitude Scale (EBPAS). *Ment Health Serv Res. 2004; 6*(2), 61–74. Retrieved from http://www.ncbi.nlm.nih.gov/pubmed/15224451

71. Centers of Disease Control and Prevention (CDC). NHANES 2009-2010. 2014. Retrieved June 23, 2017, from https://wwwn.cdc.gov/nchs/nhanes/ContinuousNhanes/Default.aspx?BeginYear=2009

72. Nelson, S, Slusar, MB, Albert, JM, Liu, Y, Riedy, CA. Psychometric properties of a caregiver illness perception measure for caries in children under 6 years old. *Journal of Psychosomatic Research. 2016; 81*, 46–53. https://doi.org/10.1016/j.jpsychores.2016.01.002

73. Morris, NS, MacLean, CD, Chew, LD, Littenberg, B. The Single Item Literacy Screener: evaluation of a brief instrument to identify limited reading ability. 2006, March 24. https://doi.org/10.1186/1471-2296-7-21

74. Nasseh K, Vujicic M. The Impact of Medicaid Reform on Children's Dental Care Utilization in Connecticut, Maryland, and Texas. Health Serv Res. 2015 Aug;50(4):1236-49. doi: 10.1111/1475-6773.12265. Epub 2014 Dec 7. PMID: 25483733; PMCID: PMC4545356.

75. Lee W, Spiekerman C, Heima M, et al. The effectiveness of xylitol in a school-based cluster randomized clinical trial. *Caries Res*. 2015; 49(1):41-49. doi:10.1159/000360869.

76. McCullagh P and Nelder JA. Generalized Linear Models, 2nd Edition. 1989. Chapman & Hall, New York, NY.

77. Morel, JG, Bokossa, MC, Neerchal, NK. "Small Sample Correction for the Variance of GEE Estimators," *Biometrical Journal*. 2003; 4, 395–409.

78. Albert, JM, Wang W, Nelson S. Estimating overall exposure effects for zero-inflated regression models with application to dental caries. *Statistical Methods in Medical Research*. 2014; 23, 257-278. Published online, September 8, 2011 as doi:10.1177/0962280211407800.

79. Albert, JM, Nelson, S. Generalized causal mediation analysis. *Biometrics. 2011; 67*(3), 1028–1038. https://doi.org/10.1111/j.1541-0420.2010.01547.x

80. Albert, JM, Cho, J, Liu, Y, & Nelson, S. (n.d.). Generalized Causal Mediation and Path Analysis: Extensions and Practical Considerations. *Unpublished Paper*.

81. Cho, J, Albert, JM. (n.d.). An R Package for Generalized Causal Mediation and Path Analysis: Extensions and Practical Considerations. *Unpublished Paper*.
