# Supplementary material for: Multilevel Interventions and Dental Attendance in Pediatric Primary Care: A Cluster Randomized Clinical Trial
Source: JAMA Netw Open. 2024 Jul 9;7(7):e2418217. doi: 10.1001/jamanetworkopen.2024.18217 (PMC11234234; doi:10.1001/jamanetworkopen.2024.18217)
Supplement: Supplement 2. — eTable 1. Details of Multi-Level Intervention eTable 2. Assumptions for Coding the Medicaid Claims Data eTable 3. Differences Between Participants and Non-Participants From Clinical Data (Baseline to WCV3) eTable 4. Differences Between Participants and Non-Participants From Medicaid Data (Baseline to 3 Years) [file jamanetwopen-e2418217-s002.pdf]

Supplemental Online Content

Nelson S, Albert JM, Selvaraj D, et al. Multilevel interventions and dental attendance in pediatric primary care: a randomized clinical trial. *JAMA Netw Open*. 2024;7(7):e2418217. doi:10.1001/jamanetworkopen.2024.18217

eTable 1. Details of Multi-Level Intervention

eTable 2. Assumptions for Coding the Medicaid Claims Data

eTable 3. Differences Between Participants and Non-Participants From Clinical Data (Baseline to WCV3)

eTable 4. Differences Between Participants and Non-Participants From Medicaid Data (Baseline to 3 Years)

This supplemental material has been provided by the authors to give readers additional information about their work.

© 2024 Nelson S et al. *JAMA Network Open*.

eTable 1. Details of Multi-Level Intervention

Intervention Level	Description of Intervention
**Practice Level Intervention**	
EMR Changes/Questions	▪ Does the child have white or brown spots? Yes/No<br>▪ Did the child go to the dentist in the past 12 months? Yes/No<br>▪ Did the provider communicate oral health facts to caregiver? Yes/No<br>▪ Did the provider give caregiver prescription to take child to the dentist and list of Medicaid-accepting dentists in the area? Yes/No
**Provider Level Intervention**	
Provider Training	▪ Common-Sense Model of Self-Regulation theory-based Didactic Education (45 minutes) to deliver the 6 oral health facts to parents<br>a. Children can have cavities without pain (Identity).<br>b. Bacteria cause cavities. Cavity-causing bacteria can multiply in the presence of sugar and poor oral hygiene (Cause).<br>c. Baby teeth are important because bacteria from cavitated primary teeth will attack newly erupting permanent teeth (Timeline).<br>d. Untreated cavities can affect your child's daily activities and overall general health (Consequences).<br>e. Cavities are preventable with self-care strategies like routine dental visits, oral hygiene, and diet (Control).<br>f. Children should have annual dental visits starting at age 1.<br>▪ Skill Training with Standardized Patients (45 minutes)
Provider Resources to deliver the intervention	▪ Flipchart (to keep in exam room)<br>▪ Prescription (to go to dentist) together with list of Medicaid accepting dentists<br>▪ Pocket Card with the 6 facts

© 2024 Nelson S et al. *JAMA Network Open*.

eTable 2. Assumptions for Coding the Medicaid Claims Data

**General Guidelines**<br>• A dental visit was defined as the use of any dental services during a single day. A combination of Current Dental Terminology (**CDT**), Current Procedural Terminology (**CPT**), International Classification of Diseases (**ICD**) codes, and provider type and specialty was used to assign each dental visit into one of the following four mutually exclusive categories **(Restorative Visit, Emergency Room Visit, Preventative Visits at the Dentist or Preventative Visits at a Primary Care Visit)**.<br>• If there were multiple types of dental visits on the same day the visit was classified in the order listed below. For e.g, if the visit was both restorative and preventative then it would be classified as restorative. If the visit was a preventative dental service that could be done by either a dentist or a primary care provider then the provider type was used to classify the visit.
1. A **Restorative Visit** was identified as the presence of CDT codes D2000-2999 (restorative procedures), D3000-D3999 (root canal procedures), or D7000-D7999 (oral surgery procedures)
2. An **Emergency Room Dental Visit** was identified as the presence of CPT codes ("99282", "99283","99284", "99285") in combination with ICD diagnosis codes for dental disease (K00-K14)
3. **Preventive Visit at the Dentist** - If the following CPT codes were identified and none of the criteria above was met (not 1 and 2) , then the visit was coded as a preventative visit at the dentist<br>i. 'D1110','D1120' (prophylaxis)<br>ii. 'D0140' (limited oral evaluation – problem focused)<br>iii. 'D1351', 'D1353', 'D1352' (sealants)<br>iv. 'D1354', 'D1355' (protective medicament)<br>v. CPT D0200- D0399 (x-rays)<br>In addition, if the provider type or specialty was a dentist and only preventative services were rendered (not 1 and 2) then the visit was considered preventive at a dentist.
c. **Preventive Visit at a Primary Care Visit** – If the following CPT codes occurred on the same day as a WCV ("99391", "99392", "99393", "99381", "99382", "99383") and none of the prior conditions were met (not 1, 2, 3a, 3b) then the visit was considered a preventative Visit at a Primary Care Visit<br>i. 'D1206','D1208' (fluoride varnish)<br>ii. 'D0603' (caries risk assessment and documentation, with a finding of high risk)<br>iii. 'D9310' (consultation, is for diagnostic service provided by a dentist or physician other than the requesting dentist or physician)<br>iv. 'D0120', 'D0150' (oral evaluation)

© 2024 Nelson S et al. *JAMA Network Open*.

eTable 3. Differences Between Participants and Non-Participants From Clinical Data (Baseline to WCV3)

Variables		Overall (n=1023)	Participant (n= 675)	Non-Participant (n= 348)	*P* value
Study Arm, n (%)	Intervention	517 (50.5)	327 (48.4)	190 (54.6)	0.06
	Control	506 (49.5)	348 (51.6)	158 (45.4)	
Race, n (%)	Black	451 (45.6)	309 (47.2)	142 (42.5)	0.31
	White	441 (44.6)	281 (42.9)	160 (47.9)	
	Other[1]	97 (9.8)	65 (9.9)	32 (9.6)	
Gender, n (%)	Female	466 (45.6)	314 (46.5)	152 (43.9)	0.43
	Male	555 (54.4)	361 (53.5)	194 (56.1)	
Age in Months (mean (SD))		56.10 (14.03)	56.80 (14.46)	54.75 (13.07)	0.03*
Education, n (%)	<= High School[2]	450 (44.7)	299 (44.6)	151 (44.8)	0.96
	> High School[3]	557 (55.3)	371 (55.4)	186 (55.2)	
Untreated Decay (dt) at Baseline (mean (SD))		0.85 (1.82)	0.88 (1.85)	0.79 (1.76)	0.44

P values based on Chi-Square test, and t-test
*Significance at alpha <0.05
[1] Other consisting of American Indian/Alaska Native, Asian, Hawaiian/Pacific Islander, More than one race
[2] <= High School consisting of Grade 7-9, Grade 10-11, and High school diploma/GED
[3] > High School consisting of Associate's degree/Some college, College/University degree, some graduate school, and Graduate degree

© 2024 Nelson S et al. *JAMA Network Open*.

eTable 4. Differences Between Participants and Non-Participants From Medicaid Data (Baseline to 3 Years)

Variables		Overall (n=1023)	Participant (n= 872)	Non-Participant (n= 151)	*P* value
Study Arm, n (%)	Intervention	517 (50.5)	450 (51.6)	67 (44.4)	0.10
	Control	506 (49.5)	422 (48.4)	84 (55.6)	
Race, n (%)	Black	451 (45.6)	378 (44.8)	73 (50.0)	0.43
	White	441 (44.6)	383 (45.4)	58 (39.7)	
	Other[1]	97 (9.8)	82 (9.7)	15 (10.3)	
Gender, n (%)	Female	466 (45.6)	391 (44.9)	75 (50.0)	0.25
	Male	555 (54.4)	480 (55.1)	75 (50.0)	
Age in Months (mean (SD))		56.10 (14.03)	55.60 (14.01)	59.05 (13.85)	<0.01*
Education, n (%)	<= High School[2]	450 (44.7)	386 (44.8)	64 (43.8)	0.82
	> High School[3]	557 (55.3)	475 (55.2)	82 (56.2)	
Untreated Decay (dt) at Baseline (mean (SD))		0.85 (1.82)	0.87 (1.85)	0.72 (1.63)	0.35

P values based on Chi-Square test, and t-test
*Significance at alpha <0.05
[1] Other consisting of American Indian/Alaska Native, Asian, Hawaiian/Pacific Islander, More than one race
[2] <= High School consisting of Grade 7-9, Grade 10-11, and High school diploma/GED
[3] > High School consisting of Associate's degree/Some college, College/University degree, some graduate school, and Graduate degree

© 2024 Nelson S et al. *JAMA Network Open*.
