# Supplementary material for: Multilevel Interventions and Dental Attendance in Pediatric Primary Care: A Cluster Randomized Clinical Trial
Source: JAMA Netw Open. 2024 Jul 9;7(7):e2418217. doi: 10.1001/jamanetworkopen.2024.18217 (PMC11234234; doi:10.1001/jamanetworkopen.2024.18217)
Supplement: Supplement 3. — Data Sharing Statement [file jamanetwopen-e2418217-s003.pdf]

Data Sharing Statement

Nelson. Multilevel Interventions and Dental Attendance in Pediatric Primary Care. *JAMA Netw Open*. Published July 09, 2024. doi:10.1001/jamanetworkopen.2024.18217

Data
Data available: Yes
Data types: Deidentified participant data, Data dictionary
How to access data: Data will be available upon request to the corresponding author (suchitra.nelson@case.edu)
When available: beginning date: 04-01-2025

Supporting Documents
Document types: None

Additional Information
Who can access the data: Data will be available upon request and after approval of a proposal and a signed data access agreement from interested researchers
Types of analyses: For any purpose
Mechanisms of data availability: After approval of a proposal and signed data access agreement.
